# Global Conservation Significance of Ecuador's Yasuní National Park

**DOI:** 10.1371/journal.pone.0008767

**Published:** 2010-01-19

**Authors:** Margot S. Bass, Matt Finer, Clinton N. Jenkins, Holger Kreft, Diego F. Cisneros-Heredia, Shawn F. McCracken, Nigel C. A. Pitman, Peter H. English, Kelly Swing, Gorky Villa, Anthony Di Fiore, Christian C. Voigt, Thomas H. Kunz

**Affiliations:** 1 Finding Species, Takoma Park, Maryland, United States of America; 2 Save America's Forests, Washington D. C., United States of America; 3 Nicholas School of the Environment, Duke University, Durham, North Carolina, United States of America; 4 Department of Biology, University of Maryland, College Park, Maryland, United States of America; 5 Division of Biological Sciences, University of California San Diego, La Jolla, California, United States of America; 6 Department of Geography, King's College London, Strand, London, United Kingdom; 7 College of Biological and Environmental Sciences, Universidad San Francisco de Quito, Quito, Ecuador; 8 Department of Biology, Texas State University, San Marcos, Texas, United States of America; 9 TADPOLE Organization, San Marcos, Texas, United States of America; 10 School of Biological Sciences, University of Texas at Austin, Austin, Texas, United States of America; 11 Department of Anthropology, New York University, New York, New York, United States of America; 12 Leibniz Institute for Zoo and Wildlife Research, Berlin, Germany; 13 Center for Ecology and Conservation Biology, Department of Biology, Boston University, Boston, Massachusetts, United States of America; University of Zurich, Switzerland

## Abstract

**Background:**

The threats facing Ecuador's Yasuní National Park are emblematic of those confronting the greater western Amazon, one of the world's last high-biodiversity wilderness areas. Notably, the country's second largest untapped oil reserves—called “ITT”—lie beneath an intact, remote section of the park. The conservation significance of Yasuní may weigh heavily in upcoming state-level and international decisions, including whether to develop the oil or invest in alternatives.

**Methodology/Principal Findings:**

We conducted the first comprehensive synthesis of biodiversity data for Yasuní. Mapping amphibian, bird, mammal, and plant distributions, we found eastern Ecuador and northern Peru to be the only regions in South America where species richness centers for all four taxonomic groups overlap. This quadruple richness center has only one viable strict protected area (IUCN levels I–IV): Yasuní. The park covers just 14% of the quadruple richness center's area, whereas active or proposed oil concessions cover 79%. Using field inventory data, we compared Yasuní's local (alpha) and landscape (gamma) diversity to other sites, in the western Amazon and globally. These analyses further suggest that Yasuní is among the most biodiverse places on Earth, with apparent world richness records for amphibians, reptiles, bats, and trees. Yasuní also protects a considerable number of threatened species and regional endemics.

**Conclusions/Significance:**

Yasuní has outstanding global conservation significance due to its extraordinary biodiversity and potential to sustain this biodiversity in the long term because of its 1) large size and wilderness character, 2) intact large-vertebrate assemblage, 3) IUCN level-II protection status in a region lacking other strict protected areas, and 4) likelihood of maintaining wet, rainforest conditions while anticipated climate change-induced drought intensifies in the eastern Amazon. However, further oil development in Yasuní jeopardizes its conservation values. These findings form the scientific basis for policy recommendations, including stopping any new oil activities and road construction in Yasuní and creating areas off-limits to large-scale development in adjacent northern Peru.

## Introduction

The western Amazon is one of the world's last high-biodiversity wilderness areas [1], [2], a region of extraordinary species richness across taxa [3]–[9] where large tracts of intact forests remain [10], [11]. Indeed, it is still possible to walk continuously through mega-diverse forest from southern Peru to southern Venezuela—a distance of ∼2,000 kilometers—without crossing a single road. However, numerous major threats confront the ecosystems of this region—including hydrocarbon and mining projects, illegal logging, oil palm plantations, and large-scale transportation projects under the umbrella of IIRSA (Initiative for the Integration of Regional Infrastructure in South America) [12]. For example, oil and gas concessions now cover vast areas, even overlapping protected areas and titled indigenous lands [13].

**Figure 1:**
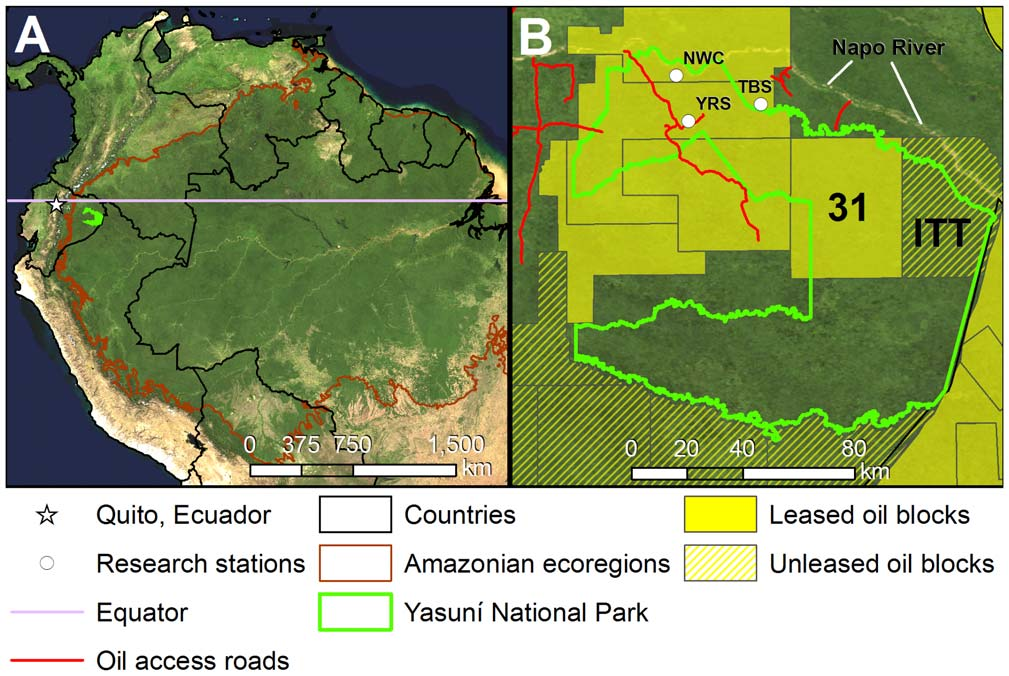
Ecuador's Yasuní National Park. A) Location of Yasuní National Park at the crossroads of the Amazon, Andes, and the Equator. B) Oil blocks and oil access roads within and surrounding the park. ITT = Ishpingo-Tambococha-Tiputini oil fields, NWC = Napo Wildlife Center, TBS = Tiputini Biodiversity Station, YRS = Yasuní Research Station. The image background is the Blue Marble mosaic of MODIS satellite images.

Yasuní National Park (Yasuní) in Ecuador is a major protected area within the western Amazon, yet it faces threats emblematic of those facing the entire region. The park occupies a unique location at the intersection of the Andes (<100 km from the Andean foothills), the Amazon (near the western phytogeographic limit of the Amazon Basin) [14], and the Equator (∼1° S) (Figure 1A). Created in 1979, Yasuní covers approximately 9,820 km^2^ [15], [16], and is surrounded by a 10 kilometer buffer zone in all directions except to the east, where it meets the Ecuador-Peru border [17]. The park overlaps ancestral Waorani (or Huaorani) territory, and is inhabited by at least two clans living in voluntary isolation [16]. In 1989, Yasuní and much of the adjacent area that is now the Waorani Ethnic Reserve were designated a UNESCO Man and the Biosphere Reserve [18]. Yasuní's climate is characterized by warm temperatures (averaging 24–27°C for all months), high rainfall (∼3,200 mm annually), and high relative humidity (averaging 80–94% throughout the year) [19]. Yasuní is within the “Core Amazon,” a particularly wet region with high annual rainfall and no severe dry season [20]. The park's elevational range is small (from ∼190 to ∼400 m above sea level), but it is crossed by frequent ridges of 25 to 70 meters [21], [22]. Soils are mostly geologically young, fluvial sediments from erosion of the Andes [22], [23]. Yasuní protects a large tract of the Napo Moist Forests terrestrial ecoregion [24] and the Upper Amazon Piedmont freshwater ecoregion, which contains numerous headwater rivers of the Amazon [25].

Several large-scale development projects exist or have been proposed within the park and its buffer zone. Leased or proposed oil concessions cover the northern half of Yasuní, and four oil access roads have already been built into the park or its buffer zone (Figure 1B). These roads have facilitated colonization, deforestation, fragmentation, and overhunting of large fauna in the northwestern section of the park [26]–[34] and illegal logging in the south and west [26], [35]. Under IIRSA, the Napo River, which borders the northern side of the park, may be dredged in order to become part of a major transport route connecting Brazil's port of Manaus with Ecuador's Pacific coastal ports [36]. Moreover, large oil palm plantations have been established near the park, just north of the Napo River. Despite these incursions, intact forest still covers the vast majority of Yasuní [32], [34].

One of the most serious issues confronting Yasuní is that Ecuador's second largest untapped oil fields lie beneath the largely intact, northeastern section of the park (in the “ITT” Block, containing the Ishpingo, Tambococha, and Tiputini oil fields; Figure 1B). The adjacent Block 31 contains additional untapped reserves underlying Yasuní. Efforts by scientists and conservationists stopped a new oil-access road into Block 31 planned by Brazil's Petrobras, but Ecuador could re-auction this block at any time. In response to strong opposition to oil drilling in Yasuní, the Government of Ecuador launched the novel Yasuní-ITT Initiative in 2007. The Initiative offers to keep ITT oil permanently underground and unexploited in exchange for financial compensation from the international community or from carbon markets [37]–[38]. The Initiative's primary goals are to respect the territory of indigenous peoples, combat climate change by keeping ∼410 M metric tons of CO_2_ out of the atmosphere, and protect the park and its biodiversity.

The global conservation significance of Yasuní—a site often referred to anecdotally as one of the most biodiverse places on Earth (*e.g.*, [39], [40])—may thus weigh heavily in upcoming state-level and international decisions affecting the park. A preliminary assessment of Yasuní's biodiversity was conducted in 2004 in response to Petrobras' planned road [27]. We build upon that effort here and provide the first comprehensive synthesis of biodiversity data for Yasuní, assessing species richness, endemism, and threatened species across various taxonomic groups. We compare our findings to those from other regions, and discuss the global conservation significance of Yasuní by evaluating its potential to sustain a high percentage of Amazonian biodiversity in the long term. We then assess the threats to Yasuní's conservation values from oil development. We close with policy recommendations drawing upon these findings.

## Results and Discussion

### Species Richness

**Figure 2:**
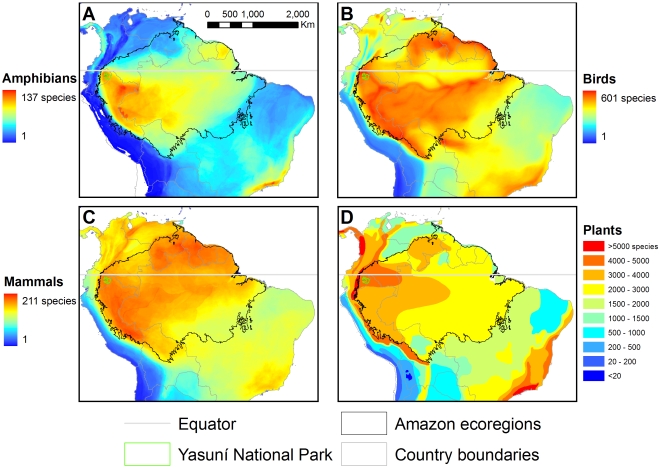
Species richness patterns of northern South America. Species richness for A) amphibians, B) birds, C) mammals, and D) vascular plants. See Materials and Methods for details.

**Figure 3:**
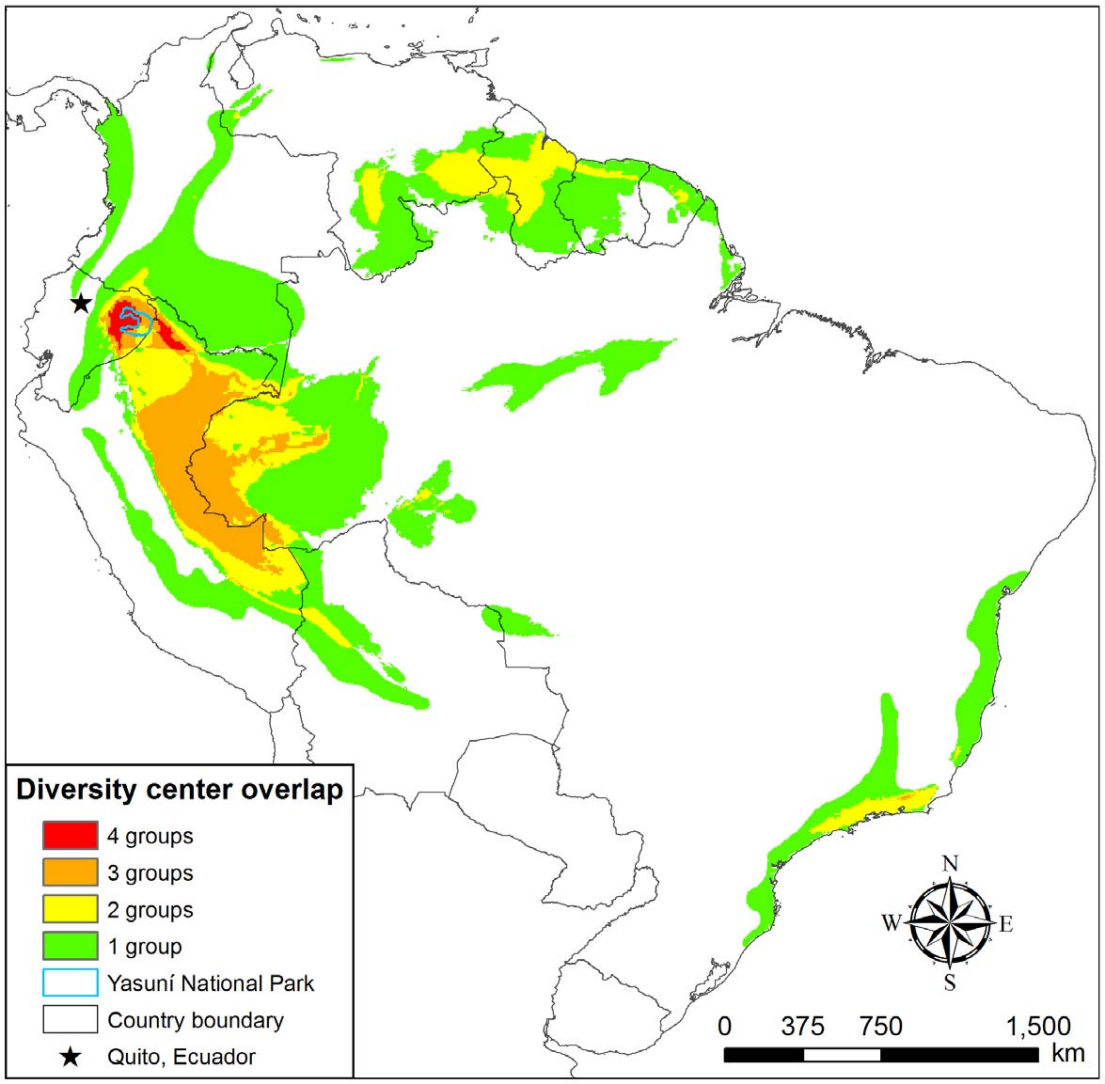
Richness center overlap. Richness center overlap of four key focus groups—amphibians, birds, mammals and vascular plants. A richness center is defined as the top 6.4% of grid cells for each taxonomic group (see Materials and Methods for details). 4 groups = area where richness centers for all four groups overlap; 3 groups = richness centers for three groups overlap; 2 groups = richness centers for two groups overlap; 1 group = richness center for just one group occurs; 0 = richness center for none of the four groups.

Distribution maps of amphibian, bird, mammal, and vascular plant species across South America (Figure 2) show that Yasuní occupies a unique biogeographic position where species richness of all four taxonomic groups reach diversity maxima (*i.e.*, quadruple richness center, see Figure 3). For amphibians, birds, and mammals, these are not just continental, but global, maxima of species richness at local scales (≤100 km^2^) [5], [41]–[43]. The same is true of tree community richness (see below). This relatively small (28,025 km^2^) quadruple richness center encompasses just 0.16% of South America and less than 0.5% of the Amazon Basin. Yasuní is the only strict protected conservation area (considered here as IUCN levels I–IV; see [44], [45]) within the quadruple richness center, covering just 14% of its area, while 79% of the center currently coincides with active or proposed oil concessions. In addition to the park, the adjacent Waorani Ethnic Reserve and a disjunct stretch just across the border in northern Loreto, Peru, account for much of the remaining area of the quadruple richness center.

To substantiate the mapping results, we synthesized data sets from field inventories and publications to establish Yasuní's “local” and “landscape” species richness. The former reflects the complexity of a community, or alpha diversity, while the latter is a measure of the total richness within an area, or gamma diversity, and is a product of the alpha diversity of its local communities and the degree of beta differentiation among them [46]. Local richness is defined here, as it is in the maps for vertebrate taxa (Figure 2A–2C), as the total species occurring in ≤100 km^2^. In the field inventories described below, local richness is typically sampled in areas ranging from a fraction of a hectare to a few hundred hectares. Landscape richness is defined here as the total number of species occurring in areas typically ≤10,000 km^2^ (after Pitman [23]), conveniently roughly equivalent to the size of Yasuní in its entirety. Species richness data qualified as “known,” “documented,” or “confirmed” refer to species actually collected, sighted, or otherwise known by experts to occur within an area. Data qualified as “expected,” “estimated,” or “projected” refer to species anticipated for an area based upon expert opinion or statistical analyses. Due to data limitations, the field inventory analyses focus more on amphibians, reptiles, birds, mammals, and vascular plants, than on fish and insects. We compare Yasuní's richness to that documented for other sites, in the western Amazon and globally. These comparisons support the mapping results, and suggest that Yasuní is among the world's most biodiverse sites, both at landscape (Table 1) and local spatial scales (Table 2).

**Table 1:** Landscape-scale species richness, threatened species, and regional endemics of Yasuní National Park.

	Species Richness^a^	Threatened Species^g^	Regional Endemics^i^
**Amphibians**	150	1	20
**Reptiles**	121	2	–
**Birds**	596	2	19
**Mammals**	169–204^b^	8	4
**Fish**	382^c^–499^d^	0	–
**Plants**	2,704^e^–∼4,000^f^	28–56^h^	∼400–720^j^

^a^Total species known for Yasuní National Park as a whole (∼10,000 km^2^), from data synthesized for this paper, unless noted.

^*b*^Lower total represents mammal species known to occur in Yasuní. Higher total is an estimate that includes species known or expected to occur in Yasuní.

^*c*^Fish species known for Yasuní [68].

^*d*^Fish species expected for Yasuní (K. Swing, unpub. data).

^*e*^Vascular species known for Yasuní (H. Mogollon and J. Guevara, unpub. data, G. Villa, unpub. data, [92]–[94]).

^*f*^Vascular plant species expected per 10,000 km^2^ in the global plant diversity center within which Yasuní lies [91].

^*g*^Total threatened species known to occur in Yasuní, including only those species listed as Critically Endangered, Endangered, or Vulnerable in the IUCN Red List of Threatened Species [47]. Data synthesized for this paper, unless noted.

^*h*^Lower total represents threatened plant species known to occur in Yasuní. Higher total is an estimate that includes threatened plant species known or expected to occur in Yasuní.

^*i*^Total regional endemics known to occur in Yasuní, from data synthesized for this paper, unless noted. Dashes indicate unknowns. See text for further description of regional endemics.

^*j*^Estimated range of total regional endemic plant species that occur in Yasuní. See text for derivation of estimates.

**Table 2:** Local-scale species richness of Yasuní National Park.

Group	No. of Species	Sample Area	Locale	Source
Amphibians	139	6.5 km^2^	TBS	[53]
Reptiles	108	6.5 km^2^	TBS	[53]
Birds	571	15 km^2^	NWC	[70]
Birds	285	1 km^2^	TBS	[74]
Birds	284	1 km^2^	YRS	[73]
Primates	10	6.5 km^2^	TBS	[80]
Bats	58	7.07 km^2^	TBS	[85]
Bats	>100 (projected)	7.07 km^2^	TBS	[85]
Trees (≥1 cm dbh)	655 (mean)	per ha (in 25 ha plot)	YRS	[96]
Trees (≥10 cm dbh)	293	1 ha	Capirón	[101]
Trees (≥10 cm dbh)	282	1 ha (in 25 ha plot)	YRS	R. Condit, pers. comm.
Trees (≥10 cm dbh)	251 (mean)	per ha (in 25 ha plot)	YRS	[96]
Trees (≥10 cm dbh)	242 (mean)	per ha (n = 19)	Within and close to Yasuní	Data taken from [101]
Epiphytes	313	6.5 km^2^	TBS	[93]
Epiphytes	146	0.1 ha	TBS	[93]
Lianas (≥1 cm)	109	1 ha (sampled with non-contiguous transects totalling 0.2 ha)	Yasuní and Waorani Ethnic Reserve	[206]
Lianas (≥1 cm)	98 (mean)	1 ha (sampled with non-contiguous transects totalling 0.2 ha) (n = 6)	Yasuní and Waorani Ethnic Reserve	[95]
Lianas (≥2.5 cm)	50	0.1 ha (sampled in non-contiguous transects, all within 1 ha plot)	Yasuní and Waorani Ethnic Reserve	[95]
Lianas (≥2.5 cm dbh)	27	0.1 ha (transect)	YRS	[92]
Lianas (all dbh size classes)	96	0.2 ha (transect)	YRS	[92]
Lianas (all dbh size classes)	65	0.1 ha (transect)	YRS	[92]

NWC = Napo Wildlife Center, TBS = Tiputini Biodiversity Station, YRS = Yasuní Research Station. No. of Species represents total species actually documented in the Sample Area through field inventories, unless otherwise noted. Tree and liana data are largely from *terra firme* forests.

The world's greatest amphibian diversity on a landscape scale is found in the upper Amazon Basin of Ecuador and Peru, and in the Atlantic Forest of eastern Brazil, according to a recent analysis reflecting distribution data and expert opinion (with richness assessed in ∼3,000 km^2^ grids) [6]. Data from field inventories support this finding. The 150 amphibian species documented to date throughout Yasuní is a world record among comparable landscapes. Yasuní's known total exceeds the IUCN database total of species known, inferred, and projected to occur in an area of similar size in the greater Iquitos region of northern Loreto, Peru (141 spp./11,310 km^2^) [47], and exceeds known field records from a much larger area sampled in that region (112 spp./∼30,150 km^2^) [48], [49]. Yasuní also tops field counts for amphibian diversity from other intensively sampled western Amazon sites: Tambopata in southern Peru (99 spp./1600 km^2^) [50] and around Leticia, Colombia (123 spp./927 km^2^) [49], [51]. The vast majority of Yasuní's species are frogs and toads (141 spp.), more than are native to the United States and Canada combined (99 spp.) [47]. At a local spatial scale, the Tiputini Biodiversity Station (TBS; see Figure 1B) currently holds the world record for amphibian alpha diversity (139 documented spp/6.5 km^2^) [52], [53]. This exceeds a recent count from Leticia, Colombia, previously described as having the richest frog assemblage in the world (98 spp./12 km straight line distance) [49], [51].

Reptile landscape richness in Yasuní is extremely high as well, with 121 species documented in the park. A smaller area just south of Iquitos is nearly as rich (120 spp./577 km^2^) [54], [55], indicating that high South American reptile landscape richness may extend across the Ecuador-Peru border between Yasuní and Iquitos. Indeed, another count in northern Loreto, Peru exceeds that of Yasuní, although for a much larger area (143 spp./∼43,425 km^2^ in the greater Iquitos region [49], [56]), with sampling throughout this area and slightly beyond (J. R. Dixon, pers. comm). By a considerable margin, Yasuní's documented landscape richness of reptiles surpasses reports for the southwestern Amazon (Tambopata, Peru: 110 spp./1600 km^2^) [50] and for all Brazilian Amazon sites except one [57], [58]. Globally, Yasuní also leads Malaysia's Kinabalu Park in number of known reptile species (112 spp./750 km^2^) [59], and although higher reports exist for Africa, they are for much larger areas [60]. To our knowledge, Samuel, Rondônia (within the Brazilian Amazon), is the only site globally with greater documented reptile richness than Yasuní's within an equivalent or smaller area (129 Squamata spp./560 km^2^) [57], [58]. That Yasuní outmatches nearly all intensively sampled sites is notable, given the limited area sampled within the park [53], [61]. At a local scale, the forests protected by Yasuní may indeed be the richest globally. TBS appears to hold the alpha diversity record for reptiles, with 108 documented species in 6.5 km^2^ [52], [53]. It greatly exceeds intensively sampled western Amazon sites to the south (*e.g.*, Cusco Amazónico in Tambopata: 89 spp./∼100 km^2^) [50], [62] and well-studied Central American localities (*e.g.*, Barro Colorado Island, Panama: 81 spp. of Squamata/3 km^2^, and La Selva, Costa Rica: 81 spp. of Squamata/15.1 km^2^) [57], [58]. Yasuní's local richness in reptile species surpasses even the richest site known in Africa (89 spp. in the 150 km^2^ Mt. Nlonako area in Cameroon) [63].

Considered together, the Yasuní herpetofauna—271 species of amphibians and reptiles—is the most diverse assemblage ever documented on a landscape scale, even higher than record totals from northern Peru (255/greater Iquitos area of ∼43,425 km^2^) [48], [49], [56] and from southern Peru (210 spp./1600 km^2^ of Tambopata) [50]. Remarkably, Yasuní harbors roughly one-third of the Amazon Basin's amphibian and reptile species, despite covering less than 0.15% of its total area (Table 3). On Yasuní's border, TBS holds the world record for local richness of known herpetofauna (247 spp./6.5 km^2^) [53], far exceeding other western Amazon localities such as those within Tambopata [50].

**Table 3:** Yasuní National Park's conservation value in terms of protecting Amazonian species.

	Yasuní^a^	Amazonia^b^	Amazonian Species in Yasuní (%)
***Area***	*9,820 km^2^*	*6,683,926 km^2^*	*0.15%*
**Amphibians**	150	527	28%
**Reptiles**	121	371^c^	33%
**Birds**	596	1,778	34%
**Mammals**	169–204	627	27–33%
**Fish**	382–499	3,200^d^	12–16%
**Plants**	2,704–∼4,000	40,000^c^	7–10%

^a^Total species known for Yasuní, from data synthesized for this paper, unless otherwise noted in Table 1.

^*b*^Unless noted, Amazonia species totals are total estimated native species defined by ecoregions [207], using maps of [208].

^*c*^Estimate from [2], compiled through literature reviews and consultations with experts.

^*d*^Fish species expected for the Amazon Basin [209].

Yasuní has high fish richness documented in some of its rivers, and may be a global center for fish landscape richness, but world-wide data are still being compiled [64], [65]. All of Yasuní's major rivers ultimately flow into the Napo River in Ecuador or Peru. The Napo River Basin is part of the Upper Amazon Piedmont freshwater ecoregion, which is considered “Globally Outstanding” because experts project its species richness and endemism to be so high [25]. The Napo River Basin has 562 fish species documented [66]. This is more than the 501 species reported for the entire Bolivian Amazon, which contains a potential hotspot of fish biodiversity [67]. The fish diversity for Yasuní includes 382 known species [68], with a total of 499 estimated (K. Swing, unpub. data). The number of known species in Yasuní alone exceeds that of the entire Mississippi River Basin (∼375 estimated spp.), one of the three largest watersheds in the world [64]. Just the lower Yasuní River Basin, in the northeast corner of the park within the ITT oil block, has 277 fish species documented [66].

The Tropical Andes contain the greatest resident bird richness on the planet at the landscape scale (as assessed in grids of ∼12,000 km^2^), but the Amazon, including Yasuní, is not far behind [42]. Remarkably, Yasuní as a whole contains at least 596 documented bird species, representing one-third of the Amazon's total native species (Table 3). At local spatial scales, a north-south stretch of forest in the western Amazon appears to be the richest known globally, whether highland or lowland. Bird lists from individual sites within Yasuní contain between 550 species (in 6.5 km^2^ at TBS) [69] and 571 species (at the 15 km^2^ Napo Wildlife Center) [70]. The only site in the world of a slightly larger, but still comparable, area where documented bird richness rivals that of Yasuní is in the southern Peruvian Amazon, where over 575 species have been found in the 50 km^2^ area around Explorer's Inn [71]. Similarly, data from standardized field plots (∼1 km^2^ and ∼15 ha) and mist netting studies indicate that local-scale bird richness within and around Yasuní [72]–[74] is rivaled only by sites in southeastern Peru [4], [69], and exceeds that of sites assessed with similar sampling methods and effort in Bolivia [72], French Guiana [75], Central America [74], [76], and other tropical areas globally, including Gabon, New Guinea, and Borneo [72].

For mammals, the Andes and eastern Africa are the richest regions in the world at the landscape scale, according to a recent analysis reflecting species distribution data and expert opinion (assessed in grids of 250,000 km^2^) [77]. Still, western Amazonian forests, including Yasuní, appear to be globally unique in their ability to support at least 200 coexisting mammal species [5]. Our Yasuní mammal list contains 169 species documented in the park, with at least 35 more expected there based on range data, for a total of 204 species. The Yasuní fauna includes approximately one-third of all Amazonian mammals (Table 3), and 44% of all mammals known from Ecuador (382 spp.) [78]. Considering that Ecuador has the world's ninth highest mammal diversity [79], finding nearly half of the country's mammals in a single park is remarkable. At a local scale, the number of coexisting mammal species is also extraordinary. Ten primate species are confirmed to coexist near TBS (A. Di Fiore, unpub. data, [80]), with two additional expected species within the park (*Saguinus nigricollis*, reported in the Block 31 oil company environmental impact assessment [81], and *Saguinus fuscicollis*, which may occur in the southwest portion of Yasuní [78]). The upper estimate of 12 primate species approaches the richest known sites in the Neotropics (14 sympatric spp. in eastern Peru and western Brazilian Amazon) [82], [83] and west Africa [84], and exceeds that for comparably-sized regions of southeast Asia. Importantly, Yasuní's primate richness represents only one major primate radiation while those in west Africa and southeast Asia represent three different primate radiations.

Yasuní has amongst the highest local bat richness for any site in the world [85]. Rigorous comparison of Yasuní's local richness with that of the Andes and Central America indicates that Yasuní has higher documented and projected richness, and is among the richest of Amazonian sites [85]. Whereas 117 bat species are estimated to occur on a regional scale within the Amazon Basin [86], Yasuní is projected to harbor comparable richness on just a local scale [85]. Using the same protocol and effort, ten plots of 1 ha within the same size area (∼7.07 km^2^) were sampled at Yasuní's TBS, at Bombuscaro River in Podocarpus National Park in Ecuador's Andes, and at La Selva in Costa Rica. (La Selva was included in the study because its bat assemblage is so well studied that it could be used to assess the accuracy of different estimation methods.) In the sample plots, documented phyllostomid species were highest in Yasuní by a statistically significant margin (TBS = 44 spp., La Selva = 31 spp., Podocarpus = 22 spp.) [85]. Using a statistical tool—Jackknife 2—to estimate total richness from the field data, phyllostomid richness was projected to be highest at TBS (TBS = 58 spp., La Selva = 39 spp., Podocarpus = 25 spp.). Rarefaction of capture data from TBS and La Selva to the number of individuals captured at Podocarpus showed that both sites were statistically richer than Podocarpus (TBS = 37±4.8 spp., La Selva = 27±3.6 spp.). Rarefaction of capture data from TBS to the number of individuals captured at La Selva showed TBS to be the richest of the three (TBS = 44±1 spp.). Capture data at TBS represented only 64% of the projected total richness. While Jackknife 2 was considered the best of the tested estimators, its projection of La Selva's phyllostomid richness was ∼20% lower than the known total. Assuming that Jackknife 2 underestimated phyllostomid richness at the other sites to the same degree (*i.e.*, ∼20% underestimate), and given the typical proportion of phyllostomids within Neotropical rainforest bat assemblages, overall bat species richness for TBS was projected to be >100 coexisting species. This was nearly double the total projected for Podocarpus (∼50 spp.) [85], and considerably more than La Selva's documented total (74 spp.) [87]. Furthermore, TBS has significantly higher diversity than the Andes or Costa Rica, as measured by both the Shannon-Weiner and Simpson diversity indices. Indeed, TBS has the highest Shannon-Weiner diversity index for any bat assemblage in the world (H′ = 3.04) [85], exceeding the global record from a savanna ecosystem in Bolivia (H′ = 2.88) [88].

For insects, global data are preliminary, but Yasuní appears to harbor extremely rich ant [40], [89] and beetle [7] assemblages. A single hectare of forest in Yasuní is projected to contain at least 100,000 insect species (T. Erwin, pers. comm.), approximately the same number of insect species as is found throughout all of North America [90]. This comparison illustrates again how extremely diverse Yasuní is at local scales. The Yasuní per-hectare insect estimate represents the highest estimated biodiversity per unit area in the world for any taxonomic group (T. Erwin, pers. comm.).

For vascular plants, Yasuní is among the richest areas globally at a landscape scale. Yasuní falls within one of only nine centers of global plant diversity, defined in a recent assessment as those areas having more than 4,000 estimated vascular plant species per 10,000 km^2^ [91]. Yasuní is not, however, in the top five richest centers (Costa Rica-Chocó, Atlantic Brazil, Tropical Eastern Andes, Northern Borneo, and New Guinea, each with more than 5,000 estimated spp./10,000 km^2^) [91]. Field inventory data lag behind that assessment, with just over 2,700 vascular plant species currently documented for Yasuní: 138 lianas [92], 313 epiphytes [93], 140 pteridophytes [94], and 2,113 trees and shrubs—with 1,813 identified (H. Mogollon and J. Guevara, unpub. data) and another 300 unidentified but morphologically distinct (G. Villa, unpub. data). Yasuní's total richness of vascular plants climbs to at least 3,213 with expected species, including 161 additional trees and shrubs collected from provinces bordering Yasuní (H. Mogollon and J. Guevara, unpub. data) and 486 lianas collected either in Yasuní or in the Waorani Ethnic Reserve [95].

At the local scale, Yasuní does appear to protect the richest area in the world of woody plant species. Yasuní holds at least four global records for documented tree and liana richness: mean number of tree and shrub species per ha (size classes ≥1 cm dbh, per 25–50 ha plot sampled) [96]; mean number of larger tree species per ha (size classes ≥10 cm dbh, per 25–50 ha plot sampled) (Tables 2 and 4, and [96]); liana species ≥2.5 cm in comparable 0.1 ha plots [92]; and liana species of all size classes in 0.1 ha plots [92]. Yasuní also holds a projected global record for tree and shrub species richness, from the Center for Tropical Forest Science (CTFS) Yasuní plot. With over 1,100 species-level taxa of trees and shrubs documented in the first 25 hectares of Yasuní's CTFS plot [96], census of the remaining 25 hectares is projected to bring the total to over 1,300 species (≥1 cm dbh) [97]. This would make it by far the richest CTFS 50 ha plot yet sampled in the world (Table 4). Yasuní also holds three world records in diversity measures for woody plant species. The Yasuní CTFS plot has the highest average diversity of trees and shrubs per ha, as measured by both the Shannon-Weiner and the Fisher's alpha diversity indices, and per 25 ha, as measured by the Fisher's alpha diversity index (with the Shannon-Weiner not available for 25 hectares across plots) (see Table 4, references therein, and [98]). At the local scale, Yasuní also holds the global lowland forest record for documented epiphytes in 0.1 ha, surpassing even some Andean counts [93]. Together, these studies suggest that a typical hectare of *terra firme* forest in Yasuní contains upwards of 655 tree species [96]—more than are native to the continental United States and Canada combined [99]—and well over 900 total species of vascular plants [92], [93], [95], [96].

**Table 4:** Global comparison of shrub and tree species richness in the Center for Tropical Forest Science (CTFS) Forest Dynamics Plots.

Site	Country	Tree Spp. (≥1 cm dbh, Mean/ha)	Tree Spp. (≥10 cm dbh, Mean/ha)	Tree Spp. (≥1 cm dbh, Total)	Fisher's alpha (Trees ≥1 cm dbh, Mean/ha)	Total Census Area (ha)	Source
Yasuní National Park	Ecuador	655	251	1,104	187.1	25	[96]
Lambir Hills National Park	Malaysia	618	247	1,182	165.3	52	[104]
Pasoh Forest Reserve	Malaysia	495	206	814	123.9	50	[210]
Khao Chong Wildlife Refuge	Thailand	–	–	612	–	24	[211]
Yunnan Province (Xishuangbanna)	China	–	–	468	–	20	[211]
Bukit Timah Nature Reserve	Singapore	276	113	329	60.0	2	[212]
Korup National Park	Cameroon	236	87	494	48.0	50	[213]
Palanan Wilderness Area	Philippines	197	100	335	43.4	16	[214]
Barro Colorado Island	Panama	169	91	301	34.6	50	[215]
Okapi Faunal Reserve (Ituri)	D.R. of Congo	161	57	420	29.5	40	[216]
La Planada Nature Reserve	Colombia	154	88	228	30.6	25	[217]
Sinharaja World Heritage Site	Sri Lanka	142	72	205	24.4	25	[218]
Doi Inthanon National Park	Thailand	104.9	66.6	162	19	15	[219]
Ken-Ting National Park	Taiwan	104	61	125	–	3	[220]
Huai Kha Khaeng W. Sanctuary	Thailand	96	65	251	23.3	50	[221]
Luquillo Experimental Forest	Puerto Rico	73.3	42.1	138	–	16	[222]
Northern Taiwan (Fushan)	Taiwan	–	–	110	–	25	[211]
Mudumalai Wildlife Sanctuary	India	24.7	19.8	71	5.9	50	[223]

The forests harboring record-setting global woody plant species richness are not restricted to Yasuní alone. While plots sampling trees and shrubs down to 1 cm dbh have not been established throughout the Amazon Basin, plots for larger trees have (≥10 cm dbh) [9], [23], [100], [101]. These confirm that the world's richest 1 ha tree plots occur in Amazonia, and that Amazonia's richest plots occupy a large east-west band of forest stretching along the equator from Yasuní to Manaus, 1,700 kilometers to the east [9], [100], [101]. Thus, the richness in tree species found in Yasuní does not extend north-south throughout all western Amazon forests [23]. It is still too early to determine which areas of this equatorial band have the most diverse tree communities at the 1 ha scale. To date, Yasuní and forests within 200 kilometers of Yasuní boast the fifth, sixth, and seventh most diverse 1 ha tree plots of the more than one hundred established in this equatorial band (Cuyabeno = 307 spp. [102], Boca Curaray = 308 spp. [101], Capirón = 293 spp.) (N. Pitman, unpub. data, [101]), exceeding all previous counts published as world records [103]. Even when compared to Malaysian forests, the richness of this narrow east-west band of Amazonian forests is apparently unmatched [104].

As can be seen from the above field data, sample areas and effort are generally not standardized throughout the tropics. We sought to address this uncertainty in three ways. First, we distinguished between “documented” species totals—*i.e.*, where identifications have been confirmed by us or other experts and thus we consider them to have minimal error—and “estimated” totals—*i.e.*, where richness numbers may be higher, but uncertainty is greater. Second, given that area has a major effect on total richness [46], [98], and that diversity in its strict sense is appropriately measured as the number of species in a sample of standard size [46], we divided our richness analyses into two area-based scales, local and landscape richness (after Whittaker [46] and Pitman *et al.* [23]). Third, we consistently noted the size of sampled areas from which richness records are drawn. When studies did not give the size, we made our own rough calculations [49]. The reader is thus alerted to any comparisons between unequal areas, providing transparency about, but not minimizing, the uncertainty of comparisons within the two area-based scales of analysis. In light of our precautions, we consider the field analyses and conclusions to be as reliable and conservative as possible (see Text S1 for more details on uncertainty in the data). With regard to the extent and boundaries of the quadruple richness center, we also must acknowledge the uneven sampling across Amazonia [5], [100], [105], [106]. Yet the most standardized and therefore definitive field inventory comparisons of Yasuní's local richness with other sites are those for trees and shrubs (Table 4, [23], [98], [100], [101]), birds [72]–[75], and bats [85]. For all these taxa, Yasuní's known or expected richness is among the highest in the world. Thus, while the boundaries and full extent of the quadruple richness center may change, the field data substantiate its general location.

### Conclusions on Yasuní's Species Richness

Yasuní National Park is globally outstanding for its exceptional biological richness on both landscape and local scales, across taxonomic groups. On a landscape scale, the area is: one of the two richest in the world for amphibian species, the second richest known to date for reptiles, within the top nine richest centers for vascular plants (and the top center for trees and shrubs), among the richest lowland areas for birds, high in mammal richness (particularly for bats), and very rich in fish species. At the local scale, species distribution maps (Figure 2) are substantiated by comparisons of field inventories, and suggest that Yasuní protects forests harboring peak global richness for amphibians, birds, and mammals. Field data further suggest that Yasuní protects the globally richest documented reptile and combined herpetofaunal communities; a large stretch of forest with the globally richest documented tree communities; a stretch of one of the globally richest documented areas for birds; and the projected globally richest bat and insect communities. Notably, the park's high species richness of different taxonomic groups does not extend uniformly north-south along the Andean foothills (Figures 2A–2D). Therefore, even within the western Amazon, Yasuní stands out.

The high landscape-scale diversity described in the Andes for some taxa is due in large part to its greater environmental heterogeneity or “geodiversity” [91]. For example, the Ecuadorian Tropical Andes have higher landscape-level plant (see Figure 2D), bird [42], and mammal diversity [77], but Yasuní is clearly richer at local scales for these three groups. At the coarse scales of analysis used in these and similar studies, typically around 10,000 km^2^, individual cells of analysis in the Andes can encompass a wide variety of habitats and environmental conditions (or even multiple mountain ranges). The consequence is to inflate the richness values well above what one would ever find at a single location on the ground. While such large biogeographic areas may indeed have the high richness numbers reported, it is unlikely that any single site within them approaches such high numbers. In Yasuní, that is not the case, as illustrated in Figure 2 by the high species richness within the finer resolution grid cells (100 km^2^) of the three animal groups—amphibians, birds, and mammals.

It is still unknown exactly why Yasuní is so diverse. Richness is likely fostered by the conditions found at this unique location at the intersection of the Andes, the Amazon, and the Equator. Pitman *et al.* [23] have speculated that the most important factors behind Yasuní's high plant diversity are the high rainfall and relatively aseasonal climate. This hypothesis is consistent with global-scale diversity trends and climate-richness relationships documented for plants and other groups of organisms (*e.g.*, [107], [108]). High annual rainfall coupled with a limited dry season appears to be a major factor for the high amphibian diversity as well [41]. Average annual rainfall in Yasuní (∼3,200 mm) is considerably higher than the average across Amazonia (∼2,400 mm) [109]. Moreover, unlike the southwestern Amazon, temperatures in Yasuní never fall below the critical plant-chilling-damage temperature of 10°C [19], [110]. This combination of ever-wet and ever-warm conditions is due to Yasuní's being at a geographic crossroads—in close proximity to both the equator and the Andes [93]. Separately, Kraft *et al.* [111] found that ecological “strategy differentiation” among species is another major factor in the maintenance of Yasuní's high tree diversity. Its aseasonality, resulting in year-round availability of fruit and flowers, may be an important factor in the park's exceptional number of coexisting birds [112] and mammals [5] and overall high animal biomass. Other potential factors abound, such as possible climatic stability over evolutionary time-scales [113], but well-supported explanations for the region's diversity are still elusive.

### Threatened Species

Yasuní is home to a considerable number of globally threatened species, *i.e.*, those listed by the IUCN as Critically Endangered, Endangered, or Vulnerable [47] (Tables 1, 5, 6, 7). These include 13 documented vertebrate species and an estimated 56 plant species (28 documented in the park, with another 28 expected). An additional 15 vertebrate species are Near Threatened, along with an estimated 47 plant species (30 documented, 17 expected).

**Table 5:** Threatened and Near Threatened species totals for Yasuní National Park.

IUCN Category	Amphibians	Reptiles	Birds	Mammals	Plants	Total
Critically Endangered (CR)	–	–	–	–	1	***1***
Endangered (EN)	–	–	–	2	4	***6***
Vulnerable (VU)	1	2	2	6	23	***34***
Near Threatened (NT)	1	–	5	9	30	***45***
**Total**	***2***	***2***	***7***	***17***	***58***	***86***

Threatened species are those listed as Critically Endangered, Endangered, or Vulnerable, while Near Threatened species are those listed as such or as the older category of Lower Risk/Near Threatened, in the IUCN Red List of Threatened Species [47]. Only species known to occur in Yasuní National Park are included in the totals.

**Table 6:** Threatened and Near Threatened vertebrates known to occur in Yasuní National Park.

Class	Family	Species	Common Name	IUCN
Amphibians	Bufonidae	*Atelopus spumarius* (complex)	Pebas Stubfoot Toad	VU
	Bufonidae	*Rhinella festae*	Valle Santiago Beaked Toad	NT
Reptiles	Podocnemididae	*Podocnemis unifilis*	Yellow-spotted River Turtle	VU
	Testudinidae	*Geochelone denticulata*	South American Yellowfoot Tortoise	VU
Birds	Psittacidae	*Ara militaris*	Military Macaw	VU
	Parulidae	*Dendroica cerulea*	Cerulean Warbler	VU
	Anatidae	*Neochen jubata*	Orinoco Goose	NT
	Accipitridae	*Harpia harpyja*	Harpy Eagle	NT
	Accipitridae	*Morphnus guianensis*	Crested Eagle	NT
	Furnariidae	*Synallaxis cherriei*	Chestnut-throated Spinetail	NT
	Thamnophilidae	*Thamnophilus praecox*	Cocha Antshrike	NT
Mammals	Mustelidae	*Pteronura brasiliensis*	Giant Otter	EN
	Atelidae	*Ateles belzebuth*	White-bellied Spider Monkey	EN
	Trichechidae	*Trichechus inunguis*	Amazonian Manatee	VU
	Tapiridae	*Tapirus terrestris*	Lowland Tapir	VU
	Dasypodidae	*Priodontes maximus*	Giant Armadillo	VU
	Atelidae	*Lagothrix poeppigii*	Poeppig's Woolly Monkey	VU
	Felidae	*Leopardus tigrinus*	Oncilla	VU
	Phyllostomidae	*Vampyressa melissa*	Melissa's Yellow-eared Bat	VU
	Callitrichidae	*Saguinus tripartitus*	Golden-mantled Tamarin	NT
	Felidae	*Leopardus wiedii*	Margay	NT
	Felidae	*Panthera onca*	Jaguar	NT
	Canidae	*Atelocynus microtis*	Short-eared Dog	NT
	Canidae	*Speothos venaticus*	Bush Dog	NT
	Myrmecophagidae	*Myrmecophaga tridactyla*	Giant Anteater	NT
	Tayassuidae	*Tayassu pecari*	White-lipped Peccary	NT
	Phyllostomidae	*Vampyrum spectrum*	Spectral Bat	NT
	Phyllostomidae	*Sturnira oporaphilum*	Tschudi's Yellow-shouldered Bat	NT

Listings in the IUCN column are from the IUCN Red List of Threatened Species [47]. Abbreviations: EN = Endangered (facing a very high risk of extinction in the wild), VU = Vulnerable (facing a high risk of extinction in the wild), and NT = Near Threatened (close to qualifying for or is likely to qualify for a threatened category in the near future).

**Table 7:** Threatened plant species known to occur in Yasuní National Park.

Family	Species	Common Names	Habit	IUCN
Annonaceae	*Rollinia helosioides*	–	Tree	CR
Apocynaceae	*Aspidosperma darienense*	–	Tree	EN
Meliaceae	*Cedrela fissilis*	Missionaries' Cedar	Tree	EN
Meliaceae	*Trichilia elsae*	–	Tree	EN
Myristicaceae	*Virola surinamensis*	Baboonwood	Tree	EN
Alismataceae	*Echinodorus eglandulosus*	–	Aquatic Herb	VU
Annonaceae	*Cremastosperma megalophyllum*	–	Tree	VU
Asteraceae	*Critonia eggersii*	–	Liana	VU
Begoniaceae	*Begonia oellgaardii*	–	Terrestrial Herb	VU
Begoniaceae	*Begonia sparreana*	–	Terrestrial Herb	VU
Dichapetalaceae	*Dichapetalum asplundeanum*	–	Tree	VU
Fabaceae *s.l.*	*Inga yasuniana*	–	Tree	VU
Gesneriaceae	*Reldia multiflora*	–	Terrestrial Herb	VU
Lecythidaceae	*Couratari guianensis*	Fine-leaf Wadara	Tree	VU
Magnoliaceae	*Talauma neillii*	–	Tree	VU
Malpighiaceae	*Bunchosia cauliflora*	–	Shrub, Tree	VU
Marantaceae	*Calathea gandersii*	–	Terrestrial Herb	VU
Meliaceae	*Cedrela odorata*	Cigar-box Wood, Red Cedar	Tree	VU
Meliaceae	*Trichilia solitudinis*	–	Tree	VU
Proteaceae	*Euplassa occidentalis*	–	Tree	VU
Rubiaceae	*Palicourea anianguana*	–	Shrub, Small Tree	VU
Rubiaceae	*Simira wurdackii*	–	Tree	VU
Sapotaceae	*Micropholis brochidodroma*	–	Tree	VU
Sapotaceae	*Pouteria gracilis*	–	Tree	VU
Sapotaceae	*Pouteria nudipetala*	–	Tree	VU
Sapotaceae	*Pouteria pubescens*	–	Tree	VU
Sapotaceae	*Pouteria vernicosa*	–	Tree	VU
Sapotaceae	*Sarcaulus vestitus*	–	Tree	VU
Annonaceae	*Rollinia dolichopetala*	–	Tree	NT
Annonaceae	*Rollinia ecuadorensis*	–	Tree	NT
Annonaceae	*Tetrameranthus globuliferus*	–	Tree	NT
Annonaceae	*Trigynaea triplinervis*	–	Tree	NT
Cecropiaceae	*Pourouma petiolulata*	–	Tree	NT
Chrysobalanaceae	*Licania velutina*	–	Tree	NT
Fabaceae *s.l.*	*Inga sarayacuensis*	–	Tree	NT
Fabaceae *s.l.*	*Senna trolliiflora*	–	Tree	NT
Gesneriaceae	*Besleria quadrangulata*	–	Subfructescent Herb	NT
Gesneriaceae	*Nautilocalyx ecuadoranus*	–	Terrestrial Herb	NT
Gesneriaceae	*Pearcea hypocyrtiflora*	–	Terrestrial Herb	NT
Lauraceae	*Nectandra microcarpa*	–	Tree	LR/nt
Loranthaceae	*Psittacanthus barlowii*	–	Parasitic Shrub	NT
Marantaceae	*Calathea paucifolia*	–	Terrestrial Herb	NT
Marantaceae	*Calathea plurispicata*	–	Terrestrial Herb	NT
Marantaceae	*Calathea veitchiana*	–	Terrestrial Herb	NT
Melastomataceae	*Clidemia longipedunculata*	–	Shrub, Small Tree	NT
Melastomataceae	*Miconia abbreviata*	–	Small Tree	LR/nt
Melastomataceae	*Miconia lugonis*	–	Tree	NT
Memecylaceae	*Mouriri laxiflora*	–	Tree	NT
Olacaceae	*Minquartia guianensis*	Black Manwood	Tree	LR/nt
Rubiaceae	*Alseis lugonis*	–	Tree	NT
Rubiaceae	*Coussarea cephaëloides*	–	Shrub, Small Tree	NT
Rubiaceae	*Coussarea dulcifolia*	–	Shrub, Small Tree	NT
Rubiaceae	*Coussarea spiciformis*	–	Shrub, Small Tree	NT
Santalaceae	*Acanthosyris annonagustata*	–	Tree	NT
Sapotaceae	*Pouteria platyphylla*	–	Tree	LR/nt
Sapotaceae	*Pradosia atroviolaceae*	–	Tree	LR/nt
Tiliaceae	*Pentaplaris huaoranica*	–	Large Tree	NT
Ulmaceae	*Ampelocera longissima*	–	Tree	NT

Listings in the IUCN column are from the IUCN Red List of Threatened Species [47]. Abbreviations: CR = (facing an extremely high risk of extinction in the wild), EN = Endangered (facing a very high risk of extinction in the wild), VU = Vulnerable (facing a high risk of extinction in the wild), and LR/nt or NT = Near Threatened (close to qualifying for or is likely to qualify for a threatened category in the near future).

The tree *Rollinia helosioides* is the only Critically Endangered species (*i.e.*, facing an extremely high risk of extinction in the wild) documented in Yasuní (Table 7). Of other plant species documented or expected in Yasuní, seven are Endangered (*i.e.*, facing a very high risk of extinction in the wild). Among these is *Cedrela fissilis*, a tree targeted by illegal loggers. Most of its natural subpopulations within Ecuador have already been destroyed [114].

Eight of the threatened vertebrates are mammals, which likely qualifies Yasuní as a threatened mammals hotspot (defined by Ceballos *et al.* [43] as being the top 5% of 10,000-km^2^ cells in a global grid). Yasuní has important populations of two globally Endangered mammal species, the White-bellied Spider Monkey (*Ateles belzebuth*) and the Giant Otter (*Pteronura brasiliensis*). The White-bellied Spider Monkey was uplisted from Vulnerable to Endangered in 2008 because it is thought to have declined by at least 50% over the past 45 years (three generations), largely due to over-hunting and habitat loss [115]. Similarly, the Giant Otter may experience a halving of population size over the next 20 years due to accelerating habitat destruction and degradation [116]. Yasuní and the Pastaza River are the Giant Otter's most important refuges in Ecuador [117]. Fewer than 250 sexually reproductive individuals are estimated to remain in-country, with Yasuní harboring an estimated 20 groups, each consisting of a reproductive pair and averaging five individuals (V. Utreras, unpub. data in [27], [117]).

Yasuní is also home to numerous globally Vulnerable species (*i.e.*, facing a high risk of extinction in the wild), including six more mammals. Poeppig's Woolly Monkey (*Lagothrix poeppigii*), Lowland Tapir (*Tapirus terrestris*), and Giant Armadillo (*Priodontes maximus*) are believed to have experienced population declines of at least 30% over the past three generations (45 years) due primarily to hunting and habitat loss [118]–[120]. Similar declines are forecast over the next several generations for the Amazonian Manatee (*Trichechus inunguis*) and Oncilla (*Leopardus tigrinus*) [121], [122]. Decline of Melissa's Yellow-eared Bat (*Vampyressa melissa*) is estimated to have been >30% over the last 10 years [123].

Yasuní contains the toad species complex *Atelopus spumarius*, currently listed as Vulnerable. This genus is experiencing drastic, widespread population declines and extinctions throughout its species' ranges in Mesoamerica and South America which are closely linked to the chytrid fungus *Batrachochytrium dendrobatidis* [124], [125]. Ron [126] predicted those areas in Ecuador most hospitable to this pathogen to be in the Andes above 1,000 m, whereas Yasuní does not extend above 400 m. However, *B. dendrobatidis* has been detected in amphibian individuals of at least eight species at lower elevations (<300 m) in the Yasuní region [127]. No epidemic-caused declines have been detected in any amphibian populations in the Yasuní area, but at least one anuran (*Leptodactylus pentadactylus*) has been observed exhibiting symptoms of chytridiomycosis, the disease caused by the *B. dendrobatidis* infection [127].

The Gran Yasuní Important Bird Area, which includes both the park and adjacent Waorani Territory, contains several rare bird species [128], including 7 species listed as Vulnerable or Near Threatened (Tables 5 and 6). The Wattled Curassow (*Crax globulosa*) has been reported, but not confirmed, for Yasuní (and thus is not included in our tallies). This species was previously known from riverine forests in eastern Ecuador [129], but may have been extirpated from the country [47]. Populations for most of the rare birds of the Gran Yasuní Important Bird Area—such as the Harpy Eagle (*Harpia harpyja*) and Crested Eagle (*Morphnus guianensis*)—are declining due to hunting pressures and habitat loss and degradation in other parts of their ranges [130], [131].

The park is also home to several species experiencing such rapid population declines that in 2008 they were Red-listed for the first time by the IUCN, as Near Threatened (*i.e.*, close to qualifying for, or likely to qualify for, a threatened category in the near future). Among these is the Golden-mantled Tamarin (*Saguinus tripartitus*), with a projected decline of around 25% over the course of three generations (18 years), due primarily to anticipated high rates of oil-related deforestation [132]. The Margay (*Leopardus wiedii*), Short-eared Dog (*Atelocynus microtis*), and White-lipped Peccary (*Tayassu pecari*) were also newly listed as Near Threatened in 2008 due to increasing threats and declining populations [47]. Due to habitat loss from deforestation, the Margay may be adequately protected only in Amazonian mega-reserves such as Yasuní [133]. Yasuní is among the most important sites in Ecuador for the Jaguar (*Panthera onca*) [33], listed as Near Threatened since 2002 [47]. In addition to the Short-eared Dog, the park harbors another canine species—the Bush Dog (*Speothos venaticus*)—that is also Near Threatened. Bush Dogs and Jaguars have been documented at TBS with camera traps (K. Swing, pers. comm.). In sum, Yasuní protects a considerable number of threatened species, and is likely a global hotspot for threatened mammals.

### Endemism

Assessing endemism in the western Amazon continues to be a major challenge. Vast areas have yet to be surveyed by scientists, and in consequence many species distributions are poorly known [100], [105], [134]. At present, better information appears to be available for amphibians and birds than for other groups. Although not generally viewed as protecting part of a region with globally outstanding endemism, Yasuní does in fact harbor a considerable number of regional endemics. It has 43 documented vertebrates and an estimated 220–720 plants (Table 1) that are regional endemics, defined here as species completely, or mostly, confined to the Napo Moist Forests ecoregion [135]. This 251,700 km^2^ area forms the northwestern part of the Napo area of endemism, one of eight such areas posited for the Amazon [136].

Yasuní is home to 20 amphibian species that are endemic to the Napo Moist Forests (Table 8), including two *Pristimantis* species endemic to the park. This number may rise, as 13 species discovered at TBS are new to science [53]. An additional 21 species have the vast majority of their ranges within the Napo Moist Forests, including the Near Threatened *Rhinella festae*. Duellman [137] indicated that the upper Amazon Basin in Ecuador and Peru is notable for its high amphibian endemism.

**Table 8:** Regionally endemic amphibians, birds, and mammals of Yasuní National Park.

Class	Species	Common Name
Amphibians	*Allobates insperatus*	–
	*Allobates zaparo*	Zaparo Poison Frog
	*Rhaebo* sp. nov. 1 (cf. *glaberrimus*)	–
	*Ameerega bilinguis*	Ecuador Poison Frog
	*Hyloxalus sauli*	Santa Cecilia Rocket Frog
	*Hyloxalus* sp. nov. 1 (*cf. bocagei*)	–
	*Hylomantis hulli*	–
	*Osteocephalus alboguttatus*	Whitebelly Treefrog
	*Pristimantis achuar*	–
	*Pristimantis aureolineatus*	–
	*Pristimantis kichwarum*	–
	*Pristimantis librarius*	–
	*Pristimantis orphnolaimus*	Lago Agrio Robber Frog
	*Pristimantis paululus*	Amazon Slope Robber Frog
	*Pristimantis pseudoacuminatus*	Sarayacu Robber Frog
	*Pristimantis* sp. 2	–
	*Pristimantis* sp. 3	–
	*Pristimantis* sp. 4	–
	*Pristimantis waoranii*	–
	*Bolitoglossa equatoriana*	Ecuador Mushroomtongue Salamander
Birds	*Mitu salvini*	Salvin's Curassow
	*Aramides calopterus*	Red-winged Wood-Rail
	*Geotrygon saphirina*	Sapphire Quail-Dove
	*Phaethornis atrimentalis*	Black-throated Hermit
	*Leucippus chlorocercus*	Olive-spotted Hummingbird
	*Galbula tombacea*	White-chinned Jacamar
	*Nonnula brunnea*	Brown Nunlet
	*Thamnophilus praecox*	Cocha Antshrike
	*Epinecrophylla fjeldsaai*	Yasuní Antwren
	*Myrmotherula sunensis*	Rio Suno Antwren
	*Herpsilochmus dugandi*	Dugand's Antwren
	*Gymnopithys lunulata*	Lunulated Antbird
	*Grallaria dignissima*	Ochre-striped Antpitta
	*Hylopezus fulviventris*	White-lored Antpitta
	*Poecilotriccus calopterus*	Golden-winged Tody-Flycatcher
	*Tolmomyias traylori*	Orange-eyed Flycatcher
	*Heterocercus aurantiivertex*	Orange-crested Manakin
	*Cacicus sclateri*	Ecuadorian Cacique
	*Ocyalus latirostris*	Band-tailed Oropendola
Mammals	*Lophostoma yasuni*	Yasuní Round-eared Bat
	*Sphiggurus ichillus*	Streaked Dwarf Porcupine
	*Saguinus tripartitus*	Golden-mantled Tamarin
	*Pithecia aequatorialis*	Equatorial Saki

Regionally endemic amphibians and mammals are restricted to the Napo Moist Forests ecoregion [135]. Birds are restricted to Upper Amazon-Napo lowlands Endemic Bird Area or otherwise noted as regionally endemic by Ridgely and Greenfield [129]. Amphibian common names are from [224]. Only species known to occur in Yasuní National Park are included in the list.

Yasuní lies within the Upper Amazon-Napo lowlands Endemic Bird Area [138]. Six of the ten range-restricted birds listed for this Endemic Bird Area are confirmed for Yasuní, including the Near Threatened Cocha Antshrike (*Thamnophilus praecox*). Ridgely and Greenfield [129] consider an additional 16 bird species to be endemic to eastern Ecuador and adjacent northeastern Peru, of which 13 are confirmed for Yasuní. Thus, at least 19 regionally endemic birds inhabit the park (Table 8).

At least four mammal species within Yasuní are endemic to the Napo Moist Forests ecoregion (Table 8). Two of them—Yasuní's Round-eared Bat (*Lophostoma yasuni*) and Streaked Dwarf Porcupine (*Sphiggurus ichillus*)—are endemic to the Ecuadorian Amazon [78]. In fact, the only known specimen of *L. yasuni* was collected inside the park [78], [139]. The Golden-mantled Tamarin and Equatorial Saki (*Pithecia aequatorialis*) cross over into Peru, but appear to be restricted to the Napo Moist Forests ecoregion [132], [140]. Yasuní is the only protected area for the Near Threatened Golden-mantled Tamarin. Adequate data on bats and rodents in this region are not available to indicate whether it is a center of endemism for mammals overall.

Given the park's extremely high plant richness, there is potential for a high number of regional plant endemics. Five species documented in Yasuní National Park have not been found anywhere else in the world: two herbaceous plants in the Begonia family, *Begonia oellgaardii* and *Begonia sparreana*; another herb, *Tiputinia foetida* (Thismiaceae), representing a new genus that lacks chlorophyll; and two trees, *Tetrameranthus globuliferus* (Annonaceae) and *Mouriri laxiflora* (Memecylaceae) (Table 7 and [141]). In addition, dozens of plant collections from the park represent species new to science that experts have not yet named, and that may not have been collected elsewhere. Kreft *et al.* [93] found that at least 10% of the 313 vascular epiphytes in Yasuní are endemic to the upper Napo region. Balslev [142] provides another estimate for regional plant endemism. His study examined distribution patterns of plants that occur in Ecuador, and sampled plants representing various life histories and taxonomic families that had both accurate distribution and altitudinal data (n = 536). Included were 128 species known to occur in the Ecuadorian Amazon. Of these, 18% (23 spp.) were endemic to an area larger than, but overlapping with, the Napo Moist Forests ecoregion. Interestingly, Pitman *et al.* [101] documented an abrupt shift in tree community structure at the genus level near the Ecuador-Peru border, so tree communities in Yasuní are distinct from those in adjacent Peru. Together, these studies suggest that there are roughly ∼400–720 regional endemic plant species in Yasuní (10%–18% endemism rate [93], [142] ×4,000 estimated plant species in 10,000 km^2^ in the plant richness center encompassing Yasuní [91]).

The total number of regionally endemic vertebrate species protected within Yasuní is not high compared to the numbers found in “biodiversity hotspots”—areas prioritized for conservation because of their endemism and vegetation loss [1]. However, the higher estimate for regionally endemic plant species protected in the park is just under 50% of the first threshold that qualifies an area as a biodiversity hotspot. The preliminary data are notable, given Yasuní's small size relative to most of the biodiversity hotspots, and suggest that the Napo Moist Forests may be globally outstanding for plant endemism. Furthermore, Yasuní is the only stable national park that is currently protecting these regional endemics (see below).

### Yasuní's Additional Conservation Values

Yasuní National Park is one of the most biodiverse places on Earth, whether assessed on a landscape or local scale, particularly for amphibians, reptiles, birds, bats, and trees. Part of this high diversity stems from a considerable number of threatened species, particularly mammals, and of regionally endemic amphibians, birds, and likely plants as well. What makes Yasuní even more special is the potential to sustain this biodiversity in the long term due to its 1) large size and wilderness character, 2) intact large-vertebrate assemblage, 3) IUCN level-II protection status in a region lacking other strictly protected areas, and 4) likelihood to maintain wet, rainforest conditions as climate change-induced drought intensifies in the eastern Amazon. In the following paragraphs, we elaborate on each of these qualities in turn.

Peres [143] argues that large (at least 10,000 km^2^) reserves connected to relatively intact surrounding landscapes are key to maintaining Amazonian biodiversity long-term (labeling these “mega-reserves”). Similarly, Mittermeier *et al.* [2] establish the unique global conservation value of areas meeting two criteria—high endemism and intactness (>10,000 km^2^ in area, >70% intact, <5 people/km^2^)—and label these “high biodiversity wilderness areas.” Assessing Yasuní under these criteria, the park protects nearly 10,000 km^2^ of forest within Amazonia, one of only five high biodiversity wilderness areas [2]. Moreover, the park is still surrounded by mostly intact forest, particularly to the south in Ecuador and to the east into Peru. To the west, the park is adjacent to the ∼6,000 km^2^ Waorani Ethnic Reserve, also generally intact. Yasuní encompasses the eastern portion of ancestral Waorani Territory, and has a relatively low (though growing) human population density, with mostly indigenous populations as inhabitants and neighbors [16], [144]. Thus, Yasuní retains all mega-reserve and wilderness characteristics, due to its large size, intact core area, largely intact surrounding forests, location within a high endemism region (Amazonia), and small human population.

Yasuní is most likely large and intact enough to accommodate viable populations of virtually all of its large or wide-ranging vertebrates. Although hunting is becoming unsustainable along the oil access roads and major rivers [26], [28], [30], [31], [33], [34], [145], the majority of the park's forest is probably still home to a largely intact assemblage of top predators, seed dispersers, herbivores, and seed predators [31], [33]. For example, preliminary analyses of five years of camera-trap data at TBS show top predators to be abundant and diverse in northern Yasuní (K. Swing, pers. comm.). Densities of jaguars in the forest at this research station appear to be amongst the highest documented in the literature, and five feline and two canine species coexist there (K. Swing, pers. comm.). Apart from providing another argument for Yasuní's extraordinary conservation value, the park's intact large-vertebrate assemblage increases its ability to protect plant and animal communities over the long term. For instance, species such as Woolly and Spider Monkeys are important seed dispersers in Yasuní for more than 200 species of tropical trees (A. Link and A. Di Fiore, unpub. data; [146]); for some large-seed species they are the only dispersers (A. Link and A. Di Fiore, unpub. data). Elsewhere in the Amazon Basin, hunting of the large vertebrates responsible for these functions (*e.g.*, jaguars, large primates, tapirs, and peccaries) is thought to be driving insidious, long-term changes in the composition and structure of plant communities, even in the absence of deforestation [147], [148].

**Figure 4:**
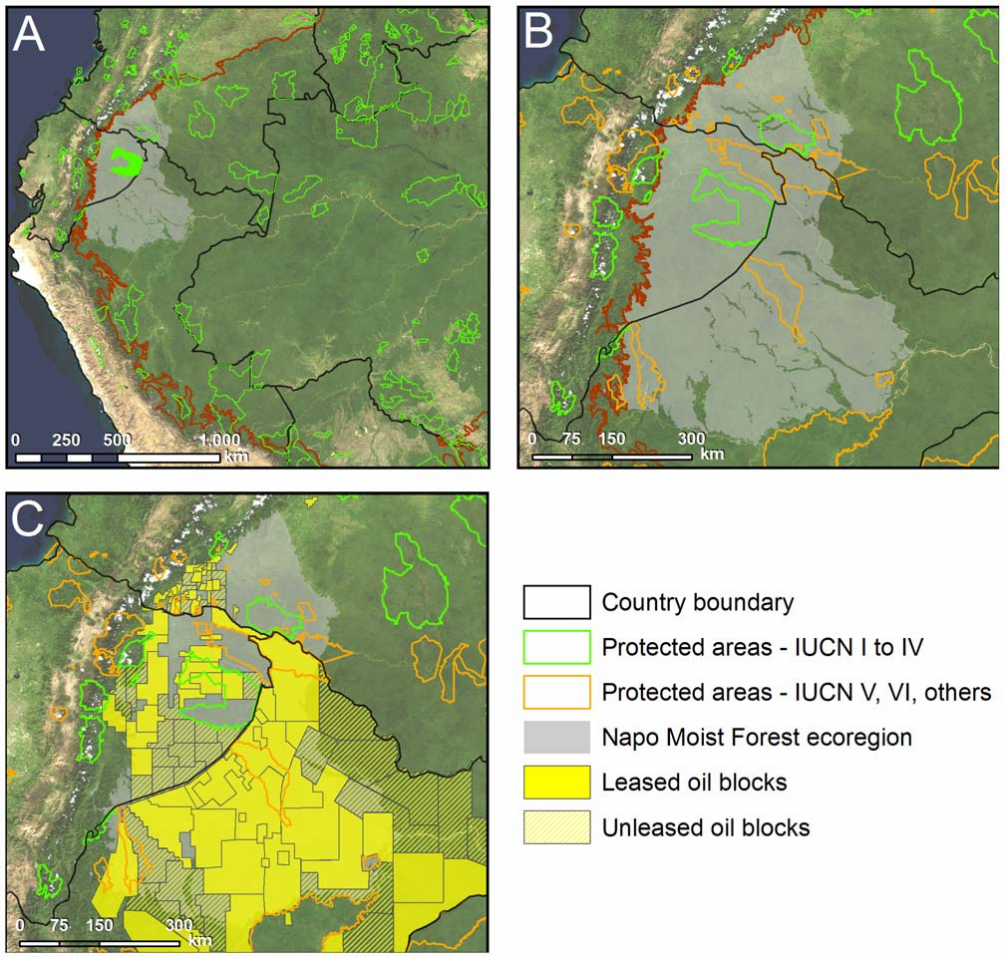
Overview of protected areas and oil blocks located within the greater Napo Moist Forest ecoregion. A) Strict protected areas (IUCN categories I–IV) in the western Amazon. B) All protected areas within the Napo Moist Forests ecoregion. C) Oil blocks covering the Napo Moist Forests ecoregion.

Yasuní is also a “lonely” park. It is currently the only strict protected area (considered here as IUCN levels I–IV) in the region capable of protecting the biodiversity of the Napo Moist Forests (Figure 4A). The only other national park fully within the Napo Moist Forests ecoregion is La Paya Natural National Park in Colombia, which is less than half the size of Yasuní, and is experiencing slash-and-burn agriculture, cattle grazing, overexploitation of aquatic fauna, illegal hunting and trapping of wildlife [149], and clearing for illicit drug crops [150]. To the west of Yasuní are the foothills of the Andes, where the species composition changes substantially. Also to the west, the Huaroani Ethnic Reserve has no specific protected area designation, and while a legal Annex to its designation specifies that activities be restricted to subsistence ones, it paradoxically requires inhabitants not to interfere with hydrocarbon exploration or exploitation [17]. To the south, the closest national park (Cordillera Azul) is more than 500 kilometers away and comprises mostly high-elevation forest rather than lowland moist forest. To the east, there is not a single strict protected area in all of northern Peru, although two areas are “reserved” but not designated for national protection (Güeppi and Pucacuro Reserved Zones) (Figure 4B), and several areas are proposed for regional-level conservation. The protection actually afforded Yasuní under the title of “national park” is in some respects only on paper, as exemplified by the extensive, ongoing oil extraction activities and permitted oil access roads. Still, the government's management plan for the park reflects its IUCN Category II designation [151], and on-the-ground biodiversity conditions appear to be much better within the park than in areas directly adjacent [29], [31], suggesting that its legal designation has significant conservation value.

Furthermore, Yasuní may serve as a refuge for Amazonian species responding to climate change. The western Amazon, unlike its eastern counterpart, has a high probability of maintaining relatively stable climatic conditions in the coming decades [20], [152]–[155]. Increased drought conditions during the dry season may be the most critical consequence of climate change in the Amazon [155], and climate models indicate a much higher probability of dry season intensification in the eastern than in the western Amazon [155]. Increased drought in the east may favor a shift from rainforest to seasonal forest, whereas the northwest Amazon is likely to maintain rainforest conditions [109]. Much of the Amazon, particularly the central region, may experience “novel” climatic conditions by the end of this century [156], conditions for which there is no contemporary counterpart. In contrast, the high precipitation in the western Amazon is controlled by regional factors (*e.g.*, the Andes forming a barrier to westward-moving moist air) that are not expected to disappear under any climate change scenario yet proposed [20], [154]. Indeed, the Napo Moist Forest region may have maintained relatively wetter conditions during dry climatic periods in the past [157]. Because of their projected climatic stability, Miles *et al.* [152] found that the forests of the western Amazon could potentially serve as a refuge for populations of the moist forest plant species of the Amazon, a large percentage of which they predict will become “non-viable” elsewhere. Furthermore, climate change is expected to push tropical species ranges upslope [158], and thus corridors are needed to facilitate migration and range shifts [20]. Recognizing those factors, Miles *et al.*'s [152] central conclusion was that, to ensure the greatest resilience of Amazonian biodiversity, the highest priority should be given to strengthening and extending protected areas in western Amazonia that encompass lowland and montane forests. In that context, Yasuní has unique value. It not only protects a lowland forest, but also, given its proximity to the Andes, could also serve as a key “stepping-stone” for climate-change driven species migrations between the Amazon forests and upslope forests found in Sumaco, Llanganates, and Sangay National Parks. Still, protected area corridors would be needed between Yasuní and these parks to allow upslope migrations.

### Threats to Yasuní's Conservation Values

Despite its being a “strict” protected area, current and pending oil projects in Yasuní threaten all four of the key strengths outlined above. Ecuador is a small nation that relies on the oil industry for half of its total export earnings and for over one-third of its annual federal budget [159]. Three fields in Yasuní—Ishpingo-Tambococha-Tiputini—contain ∼850 million barrels of crude oil, or ∼20% of Ecuador's known reserves (the ITT Block; Figure 1B). In addition, adjacent Block 31 has significant reserves that could be developed with the potential for sharing ITT infrastructure. Thus, pressure to drill in ITT has understandably been intense.

In that context, the announcement of Ecuadorian President Rafael Correa in June 2007 to postpone ITT drilling plans and seek an alternative way forward was very progressive. Ecuador has calculated that government earnings from exploitation of ITT's crude oil are roughly equivalent to the carbon market value of the oil, both around $7 billion [38]. Furthermore, the total value to Ecuador of pursuing the Yasuní-ITT Initiative is considerably higher, even from a strictly economic point of view. By precluding new oil production infrastructure and access routes, the Yasuní-ITT Initiative would help keep forests in this region intact, generating benefits through maintenance of forest carbon, ecosystem services, and biodiversity. Although no market valuation exists specifically for Yasuní, recognized economists, including Robert Costanza, have established that standing tropical forests offer significant financial value. The Yasuní-ITT Initiative will generate economic benefits even beyond the park. Ecuador plans to invest the carbon monies it receives from the Initiative not only in the management and conservation of Yasuní, but also in the country's entire protected area network (SNAP) and indigenous territories, and in other conservation and sustainable development projects [38]. SNAP includes lands already prioritized as globally valuable investments for conservation dollars, including sizeable portions of the Tropical Andes and Tumbes-Chocó-Magdalena hotpots [1], [160]. The Ecuadorian government now has a high-level team developing and promoting the Yasuní-ITT Initiative, making Ecuador's revolutionary initiative a viable proposal on the international stage.

Explicit in the messaging of the Yasuní-ITT Initiative was the recognition of the potential threats oil production could pose to the biodiversity of the region. Threats come from both direct and indirect impacts [13], [27], [161], [162]. Direct impacts of oil development include immediate deforestation for the project's production plant, drilling platforms, access routes, and pipelines, along with contamination from any project-related spills, leaks, or accidents. A preliminary study of potential environmental impacts from exploiting the ITT oil fields, conducted in 2007 by Ecuador's state oil company, Petroecuador, revealed that direct impacts would likely be substantial. According to this report, the project would require a major processing facility (∼6 ha), seven separate platforms (six for production and one for reinjection), and a new rail system to access these platforms, which would be spread along the entire length of the ITT block [163]. Oil-related contamination threatens Yasuní's large aquatic mammals, such as the Endangered Giant Otter and the Vulnerable Amazonian Manatee [116], [122]. Both species have been documented in the Tiputini and Yasuní Rivers [117], [164], which would likely be the principal access routes and infrastructure sites for oil development in ITT or the adjacent Block 31.

Compared to the direct impacts, the indirect impacts of new oil development in ITT or Block 31 are likely to be even greater: colonization and its subsequent secondary deforestation, fragmentation, and unsustainable hunting and fishing. All would intensify biodiversity loss. As indicated above, preliminary ITT development plans call for an extensive new transport and pipeline infrastructure. While plans reference train access, companies are much more likely to seek permits for building new roads, the most widespread and proven method of accessing land-based oil reserves. In either case, there would be unprecedented human access to one of the most intact portions of the Ecuadorian Amazon [13].

Indeed, oil development and its indirect impacts have played a major role in turning the Napo region into one of the 14 major deforestation fronts in the world [165]. Ecuador has had the highest deforestation rate of any Latin American country for several years [166], [167]. Wunder [168] discussed how oil development typically decreases overall deforestation in a region, largely by reducing pressure from agricultural and logging interests. However, Ecuador was shown to be the primary exception to this phenomenon, mainly because the oil itself was located deep in primary forest and the extensive system of oil access roads opened the forest [168]. Access facilitated colonization and subsequent deforestation by small-scale migrant farmers pursuing agriculture and cattle ranching [168]–[174], with an additional role played by indigenous peoples' farming of commercial crops [174].

Prior to intensification of oil exploration in the 1970s, the total deforested area in the Ecuadorian Amazon was only ∼410,000 hectares (data [171], synthesized in [172]). Only 4.1% of the forests were within 5 kilometers of a road [174], the maximum distance for the practice of successful agriculture [175]. From 1986 to 2001, concentrated oil exploitation in northeastern Ecuador—with attendant in-migration, farming, and urbanization—resulted in deforestation averaging 40,000 hectares per year [172], [174]. For each kilometer of road constructed, ∼120 hectares of agricultural lands have been cleared [174]. Unlike Brazil, agricultural lands in the Ecuadorian Amazon do not appear to be abandoned over time, but remain in use by colonists even as more areas are cleared [174]. By 2001, nearly 33% of the Ecuadorian Amazon was within 5 kilometers of a road [174]. Researchers have concluded that oil exploration, production, and associated road construction programs by the oil industry and the government are responsible for this fast-paced deforestation [168], [176].

Within Yasuní, on-the-ground impacts from oil development have diverged from oil company intentions and their projections in Environmental Management Plans. Social conditions and pressures have affected the Plans in ways difficult to address. For example, the U.S.-based Maxus oil company sought innovations to control environmental impacts when developing Block 16 in Yasuní. From 1992 to 1993, the company constructed a 150-kilometer road—the Via Pompeya Sur-Iro or informally “Via Maxus”—from the Napo River's southern shore, through Yasuní, and ending in the Waorani Ethnic Reserve [30], [177], [178]. However, Maxus did not build a bridge connecting this road to Ecuador's highway network [178], as Texaco had done when constructing the nearby Via Auca in the 1980s [179]. The Via Auca starts in Puerto Francisco de Orellana (El Coca) with a bridge crossing the Napo River and ends in Waorani territory, and has been associated with extensive environmental and social change [26], [179]. In contrast, to reach the Via Maxus, all trucks and equipment must cross the Napo River on barges [178]. The corporate intent was that this logistical obstacle to outsider vehicles and migrants would limit access, and thereby avoid colonization and secondary deforestation in the park [178].

In addition, the company's Environmental Management Plan called for numerous controls on colonization, deforestation, and hunting [180]. For example, by placing the pipeline underground and by using an innovative “geogrid” plastic to stabilize the roadbed, deforestation would be reduced in two ways [177]. The right-of-way would be narrowed to 25 meters instead of the typical 60 meters, and the clearing to provide logs to stabilize the roadbed would be reduced by 70% compared to the extent typically lost for tropical road construction [180]. Remarkably, the Plan stated that the total area deforested for the Via Maxus, the secondary roads, and all installations would be only 400 hectares (4 km^2^) [180]. Checkpoints and ground patrols would control colonization, and high-resolution satellite imaging would be used regularly to verify control [180]. Corporate officials and contractors would be prohibited from purchasing meat, fish, or other products from the Waorani [180]. Frequent audits would ensure compliance with this Plan [180].

Although most innovations were indeed implemented, environmental impacts in Block 16 in Yasuní from the Via Maxus have been significant [26], [27], [29]–[31], [34]. The road has attracted indigenous migration and building of new villages near and within the park [27], [29], [30]. Deforestation has resulted, estimated at a rate of 0.11% per year, with that rate increasing over the years [29]. Proximity to the Via Maxus is the strongest spatial factor in predicting where deforestation is occurring [29]. A conservative model based on these data projects that 50% of the forest within two kilometers of the Via Maxus will be deforested by 2063 due to settlements and forest conversion [29]. That projected area would be at least 148 km^2^ and 37 times greater than what Maxus had stated would be deforested in its Environmental Management Plan. Although forest loss is better controlled within the park than outside it [29], [172], it is undermining Yasuní's conservation values as a strict protected area and as a potential refuge for species migrating due to climate change.

Oil development and resulting impacts also threaten Yasuní's wilderness characters and its largely intact mega-faunal assemblage. The Via Maxus fragmented the northwestern section of Yasuní from the rest of the park. Further fragmentation is occurring because the Via Auca is facilitating illegal logging in Yasuní [26], [35]. Irreversible impacts on the park's biodiversity may occur even faster from fragmentation than from deforestation, based on regional analyses [172], [174]. Large predator species may need unfragmented forest areas as large as 1 million hectares to persist [181]. Rare species, such as the Near Threatened Jaguar, Margay, Short-eared Dog, and Golden-mantled Tamarin, are also susceptible to the effects of oil-industry-related deforestation and fragmentation [132], [133], [182], [183].

The Via Maxus and transport provided by oil companies to indigenous hunters are facilitating increased hunting in Yasuní [30], [31], [33], [34], [145]. Although indigenous populations have hunted in this region for generations, there is evidence that hunting is now disrupting populations of large, keystone vertebrates. Local depletion of the Endangered White-bellied Spider Monkey (*Ateles belzebuth*) has been documented along the road [30], and modeling of field takes by indigenous communities living along the road indicates that hunting of this primate is unsustainable, along with that of four other species: Red Howler Monkey (*Alouatta seniculus*), White-fronted Capuchin (*Cebus albifrons*), White-lipped Peccary (*Tayassu pecari*), and Poeppig's Woolly Monkey (*Lagothrix lagotricha*) [28], [145]. A study from February 2005 to March 2006 registered 40% lower mammal abundance along the Via Maxus compared to a control area in intact forest distant from roads [31]. A new camera-trapping study is providing similar results [33]. At least 47 species of wildlife—mostly mammals and fish, but also birds and reptiles—have been sold by indigenous hunters at a new market at the entrance of this oil access road [34]. In sum, hunting is diminishing Yasuní's conservation value in supporting an intact large-vertebrate assemblage. Also, given that many of the targeted large vertebrates are important seed dispersers, hunting could, over time, diminish Yasuní's conservation value in maintaining animal and plant composition and diversity (A. Di Fiore and A. Link, unpub. data, [31], [147], [148]).

Clearly, impacts from oil development in this region cannot be fully controlled [27], irrespective of corporate intentions and innovations. These direct and indirect impacts have the potential to be region wide, as active and proposed oil blocks blanket not just much of eastern Ecuador, but virtually all of northern Peru as well (Figure 4C). A striking example from Ecuador illustrates the reality of this threat. A site known as Santa Cecilia, located just north of Yasuní, had some of the richest amphibian [41] and reptile [57] diversity in the world. This site is now completely deforested due to oil-related disturbance and colonization [172], [184].

### Implications for Conservation

Our findings on Yasuní's biodiversity, its additional conservation values, and the documented impacts from oil development regionally and in the park itself form the scientific basis for the following five policy recommendations. 1) Permit no new roads nor other transportation access routes—such as new oil access roads, train rails, canals, and extensions of existing roads—within Yasuní National Park or its buffer zone. 2) Permit no new oil exploration or development projects in Yasuní, particularly in the remote and relatively intact Block 31 and ITT Block. 3) Create protected biological corridors from Yasuní to nearby higher-elevation Andean parks for species on the move due to climate change. 4) Create a system of strict protected areas and no-go zones (*i.e.*, off-limits to oil exploration and exploitation) in the northern Peruvian Amazon. 5) Establish a protected corridor between Yasuní and Cuyabeno Wildlife Reserve that, together with the Peruvian reserves, would form a trans-boundary mega-reserve with Yasuní National Park at its core.

In regard to recommendations 4 and 5, we emphasize that Ecuador has already created two “untouchable zones” (“zonas intangibles” in Spanish) off-limits to oil activities, one in the southern part of Yasuní and the other just north of it in Cuyabeno. The former zone was created to protect Ecuador's last indigenous peoples living in voluntary isolation, and anthropological evidence indicates that they cross the border into Peru as well [185]. Thus, areas off-limits to oil activities are needed in northern Peru not only to conserve its high biodiversity [101], but the territories of indigenous peoples as well.

In closing, we reiterate the conclusions of Malhi *et al.* [155] and Killeen and Solórzano [20], that keeping the northwestern Amazon—home to the Basin's highest biodiversity and the region least vulnerable to climatic drying—largely intact as a biological refuge is a global conservation priority of the first order. If the world's most diverse forests cannot be protected in Yasuní, it seems unlikely that they can be protected anywhere else.

## Materials and Methods

We calculated the congruence of richness centers in South America for vascular plants, amphibians, mammals, and birds, the groups for which sufficient data were available. For amphibians, mammals, and birds, we used extent-of-occurrence maps. Bird data are from Ridgley *et al.* [186], mammal data from the Global Mammal Assessment [187], and amphibian data from the Global Amphibian Assessment [188]. Species presences for these three groups were summed across an equal area grid of 100 km^2^ (10 km×10 km) to generate maps of species richness. While the species richness for vertebrates could be mapped on a continuous scale, the plant richness data, obtained from Barthlott *et al.* [91], are spatially aggregated into areas having a range of species richness (*e.g.*, a spatial unit has between 1,000 and 2,000 spp./10,000 km^2^). We therefore restricted the analysis to match the form of the plant data. We defined a richness center for plants as any region containing ≥4,000 vascular plant species per 10,000 km^2^. Only nine diversity centers worldwide reach this species density (three of which are in South America) [91]. These plant species richness centers cover 6.4% of South America, close to the 5% threshold used in similar studies (*e.g.*, [189]). We used the same 6.4% area threshold to define richness centers for birds, mammals, and amphibians (*i.e.*, the richest 6.4% of all grid cells for these groups were selected). The congruence of richness centers was determined by spatially overlaying the maps for the four taxa. The maximum value of four indicates congruent richness centers for all groups investigated.

We also conducted an extensive literature review of field studies investigating the biodiversity of Yasuní National Park (Yasuní), synthesized relevant information, and then compared it to published maps and field inventory research from around the globe. Results on species richness were grouped into two spatial categories: landscape-scale richness, typically of ≤10,000 km^2^, and local^-^scale richness, of ≤100 km^2^, but generally on the order of 100 hectares to a fraction of a hectare (after Whittaker [46] and Pitman *et al.* [23]). When comparing Yasuní's landscape richness to that documented for other areas in field inventories and maps at this scale, we used species counts established for the entire park (∼10,000 km^2^), as described below. Where total size of areas sampled was lacking in published field inventories for other regions, we calculated an estimated size by mapping the given study site locations on Google Earth 5.0 [49] and using the software to create a polygon inclusive of all sites.

We compiled lists of amphibian, reptile, bird, mammal, and plant species that occur in Yasuní National Park by collating published and unpublished inventory lists. Species richness data labeled in the text as “known,” “documented,” or “confirmed” refer to species actually collected, sighted, or otherwise known by experts to occur within the boundaries of Yasuní National Park, or collected from the Tiputini Biodiversity Station (TBS) directly adjacent to the park. Data labeled in the text as “expected,” “estimated,” or “projected” refers to species not documented within the park or TBS, but anticipated to occur there based upon expert analysis of range distributions or statistical analyses. Much information is from research at the Napo Wildlife Center and Yasuní Research Station, both located within Yasuní National Park, and from TBS (see Figure 1B).

The amphibian species list was based largely on inventories carried out at TBS and the Yasuní Research Station. The reptile list was based on inventories at TBS. D. F. Cisneros-Heredia conducted herpetofaunal inventories at TBS annually from 1997 to 2001, employing the following survey techniques: visual encounter transects, leaf-litter quadrats, pitfall traps, amphibian larvae surveys, and random point sampling [52]. S. F. McCracken conducted amphibian inventories, using leaf-litter quadrat surveys, at TBS annually from 2002 to 2004, and canopy bromeliad patch sampling at TBS and the Yasuní Research Station in 2006 and 2008 (S. McCracken, unpub. data, [190]). Incidental amphibian and reptile observations recorded by D. F. Cisneros-Heredia and S. F. McCracken at TBS and by S. F. McCracken at the Yasuní Research Station were included in the amphibian and reptile species lists. Additional amphibian species records for Yasuní National Park were included from S. Ron [191]. In addition, confirmed records of reptiles and amphibians based on voucher specimens collected within Yasuní National Park and around TBS were included. For these, D. F. Cisneros-Heredia examined amphibian and reptile specimens deposited at the following herpetological collections: Museo Ecuatoriano de Ciencias Naturales (DHMECN), Universidad San Francisco de Quito (DFCH-USFQ), Fundación Herpetológica “Gustavo Orcés” (FHGO), National Museum of Natural History, Smithsonian Institution (USNM), and Universidad Católica del Ecuador (QCAZ). D. F. Cisneros-Heredia and S. F. McCracken updated the taxonomy of both lists. To generate a total of known, inferred, and projected amphibian species on a landscape scale for greater Iquitos, Peru (in 11,310 km^2^), data were extracted from IUCN Red List of Threatened Species [47].

The bird list combined tallies from the Napo Wildlife Center [70], TBS (J. C. Arvin *et al.*, unpub. data, provided by K. Swing, [69]), and studies conducted in Block 31 [192]. Habitats for the Napo Wildlife Center list included a large river (the Napo River), river islands on the southern side of the Napo, the river's edge, secondary and primary *terra firme* forest, and a clay lick. Habitats for the TBS list included *terra firme* forest, seasonally flooded forest, tree-fall gaps, the Tiputini River, and an oxbow lake. Documentation included tape recordings, photographs, sight records, auditory observations, and substantiated observations by recognized experts dating back to 1991. We counted only species for which documentation or reliable information was given. P. English reviewed and updated the taxonomy used.

The mammal list started with data from the Campos [193] list developed as part of the Ecuadorian government's Yasuní management plan, and was augmented by data from Utreras and Jorgenson [194], Tirira [78], and Rex *et al.* [85]. The entire mammal list was then reviewed by A. L. Gardner, who provided additional species for the list and classified species as known, expected, probable, possible, doubtful, or incorrect for Yasuní National Park. Further additions to the list were provided by C. C. Voigt and T. H. Kunz (unpub. data), A. Di Fiore (unpub. data, [195]) and K. Jung (pers. comm.). Taxonomy was updated and standardized to follow Wilson and Reeder [196]. Species were then counted as known for Yasuní National Park if they were: documented by Rex *et al.* [85], specifically listed as occurring in the park by Tirira [78], classified as known for the park by A. L. Gardner, and/or observed with up-close certainty in the park or at TBS by K. Rex, T. H. Kunz, or C. C. Voigt. Species were counted as expected if they were documented by Rex *et al.* [85] and/or listed as occurring in Yasuní National Park by Tirira [78], but had tentative identifications (*cf*) or were new to science (*sp. nov.*). Species were also counted as “expected” if they were listed as such for Yasuní by A. L. Gardner (pers. comm.) and not yet documented there by other reliable sources. A final review of the list was done by A. Di Fiore, C. C. Voigt, and T. H. Kunz. We consider the final list as the only current, accurate source of total known and expected mammal species for Yasuní, both because of the extensive peer review process it underwent and its updated taxonomy.

A comprehensive plant list was not compiled *de novo*. Instead, we used totals from two comprehensive lists to be published shortly (G. Villa, unpub. data, H. Mogollon and J. Guevara, unpub. data) for known and expected vascular plant species in Yasuní. We did compile and verify our own known and expected threatened plant list. The preliminary list was compiled from a list of plant species of concern in Yasuní developed for Finding Species by H. Mogollon and J. Guevara (unpub. data) and from data in Valencia *et al.* [197]. This was then augmented and corrected by G. Villa, with known and expected presence in Yasuní verified in accordance with the definitions given above, using online plant lists and collection records from Aarhus University [198], Center for Tropical Forest Science [199], Chicago Field Museum [200], Finding Species [201], Missouri Botanical Garden [202], New York Botanical Garden [203], and the IUCN Red List of Threatened Species [47]. Where species names could not be verified in ITIS [204], they were verified in Jørgensen and León-Yánez [205].

The number of expected fish species in Yasuní comes from a 1999 synthesis of publications and fish lists from Ecuador and neighboring countries by K. Swing (unpub. data).

Conservation status for all species comes from the IUCN Red List of Threatened Species [47]. To determine endemic status, mammal range maps were reviewed from Tirira [78] and from the IUCN Red List of Threatened Species [47], and amphibian range maps from only the latter source. Boundaries of protected areas used in the figures are from the online 2007 World Database of Protected Areas, developed by UNEP-WCMC and the IUCN World Commission on Protected Areas.

## Supporting Information

Text S1 Uncertainty of Species Richness Results
(0.07 MB DOC)
