# Supplementary material for: Global Conservation Significance of Ecuador's Yasuní National Park
Source: PLoS One. 2010 Jan 19;5(1):e8767. doi: 10.1371/journal.pone.0008767 (PMC2808245; doi:10.1371/journal.pone.0008767)
Supplement: Text S1 — Uncertainty of Species Richness Results (0.07 MB DOC) [file pone.0008767.s001.doc]

**Supporting Text**

*Uncertainty of Species Richness Results*

As seen in the preceeding sythesis of field data on species richness, size of areas sampled and sampling effort are not generally standardized throughout the tropics. We sought to address this uncertainty in three ways. First, we distinguished between “documented” or “known” species totals—*i.e.*, where identifications have been confirmed by us or other experts and thus we consider them to have minimal error—and “estimated” or “projected” totals—*i.e.*, where richness numbers may be higher, but uncertainty is greater. We endeavored to use these two different estimates carefully, by making comparisons that are on an equal footing wherever possible (*i.e.*, known richness of one site is compared to known richness of another, while estimated is compared to estimated).

The issue of “known” and “projected” species richness, particularly when sample areas and methods are different, is best illustrated by a comparative survey of bat local richness. In the Rex *et al.* study described above [85], plots of equal size and sample effort [85] indicate that both the *documented* (58 spp.) and *projected* (> 100 spp.) local bat richness of Yasuní surpass an Andean and a Central American site, and that the park’s richness is likely to exceed most Amazonian sites. Yet Yasuní’s total *documented* local bat richness (58 spp.) in that study falls well short of the *documented* richness of Central and South American sites sampled with different protocols, research areas, and hours of effort, including: Central Amazonia (72 spp. each at two different sites) [225,226], Central Guyana (86 spp.) [227], French Guiana (78 spp.) [228], and Barro Colorado Island in Panama (72 spp.) (E. Kalko, pers. comm. in [226]). Conversely, the *projected* local richness of Yasuní (> 100 spp.) greatly exceeds the *documented* richness of all these. Still, no inventory of the *documented* richness at these sites, with the exception of those for BCI and La Selva, is considered complete [226]. These seemingly contradictory results highlight the importance of specifying whether richness is known or projected, and whether inventories are considered nearly complete or not.

Area has a major effect on total richness [46,98], and diversity in its strict sense is appropriately measured as the number of species in a sample of standard size [46]. Our second approach to addressing uncertainty took these factors into account; we broke our richness analyses into two area-based scales, local (alpha) and landscape (gamma) richness (after Whittaker [46] and Pitman *et al.* [23]). Yasuní landscape richness totals for amphibians, reptiles, birds, and mammals are given only as conservative “known” minimums (Table 1). Because a comprehensive census of the gamma-biodiversity of Yasuní is not yet available, we had to establish our own working number for species present in ~10,000 km^2^ for these taxa. One common way to do this is to use species-area or species-individual curves based on quantitative surveys at the local scale to estimate the number of species expected to occur in the larger area, accompanied by margins of error (*e.g.*, [229]). We decided against this method for two reasons. First, the largest quantitative surveys carried out to date in Yasuní have focused on one very diverse habitat—upland or *terra firme* forest—meaning that extrapolations from those surveys could overestimate regional diversity. Second, basing our working landscape totals on the fraction of surveys in Yasuní that are quantitative would have meant disregarding the region’s wealth of qualitative surveys—perhaps dozens of person-years worth of sampling all known habitats, including thousands of observations from a 120-km linear transect transversing Yasuní (the Via Pompeya Sur-Iro, described in the main text, which had been bordered by intact forest).

Iinstead of estimating the total number of amphibian, reptile, bird, and mammal species in Yasuní by extrapolation, we synthesized different existing species lists. We then added newer reliable reports and discarded unreliable identifications/records to generate a single landscape-scale metric that we consider reliable for each of these taxonomic groups (Table 1). Every taxon we included represents an actual field observation; none is a hypothetical “extrapolated” species that may or may not be present in the area. In the case of fish and plants, we did not compile our own lists, and had two metrics available for each—“known” and “expected” totals. The known totals for Yasuní’s plant and fish richness had been established by experts in their respective fields of expertise (plants: H. Mogollon and J. Guevara, unpub. data, and G. Villa, unpub. data, and fish: R. Barriga [68]). As with our totals, their counts cover those species actually documented in the park. The differences between the “known” field inventory totals and the “estimated” totals for plants and fish are very large (Table 1). The lower “known” totals are the conservative values.

While the “known” totals for landscape diversity do not include margins of error, uncertainty does exist about these numbers. Three sources of uncertainty, small but unquantifiable, suggest that the “known” totals may be too high. First, taxonomic experts may have made mistakes in identifications of field or museum specimens, and we may have made incorrect judgments about the reliability of such identifications. Consequently, some species might be included in our Yasuní lists that do not actually occur there. Second, “known” totals will tend to decrease with any future taxonomic lumping, where taxonomic experts group together two or more previously distinct species. Third, our landscape totals rely upon lists and collections from different sections of Yasuní. While the specimens contributing to these totals were collected within an area of just under 10,000 km^2^ (the total size of the park), the collection area was horseshoe-shaped. In consequence, Yasuní’s richness numbers may not precisely correspond to what would be found within the square grid generally used in standardized landscape analyses to assess species richness. In consequence, “known” totals may potentially be inflated due to the phenomenon of beta differentiation over the additional distance extending beyond a square grid [46]. We are not aware of any standardized method to precisely quantify the potential reduction in our known totals—and thereby quantify the uncertainty—from these three sources of possible error.

At least two sources of uncertainty suggest that the “known” totals are too low: inadequate exploration and incomplete taxonomic work. Research in Yasuní has been limited both in terms of sample sizes and distribution, with research generally concentrated at three sites—the Yasuní Research Station (YRS), the Tiputini Biodiversity Station (TBS), and the Napo Wildlife Center (NWC) (Figure 1B)—all in the northwestern section of the park and extending over a total area of only ~500 km^2^ (5% of the park). Even for plants, where sampling has been most widely distributed [23,93–95,101], research has been scant in eastern and southern Yasuní. Furthermore, experts have not yet had time to identify many of the collections made in the park. In light of recent taxonomic findings, many collections are likely to represent species and even genera that are new to science. Taking all these uncertainties into account, we think that Yasuní’s gamma diversity for each taxonomic group is likely to be considerably higher than our “known” totals, and that the size of any errors will not change our primary conclusion that the gamma diversity of Yasuní is extremely high.

On the local geographic scale, we know of only three taxa—birds, trees, and lianas—where sample effort and area are consistent within Yasuní and are rigorous in their identifications, and so can be used to gauge the uncertainty of the park’s documented species richness. For birds, three *terra firme* plots of equal sample size (100 ha) and sample effort have been established: one at YRS [73], and two at TBS, with ~30 km between YRS and TBS plots, and 1.5 km between TBS plots [74]. As these were established by two different researchers, it is possible that there is observer bias, although its direction and potential severity are unknown. Documented species richness is nearly identical in the three plots (YRS = 284 spp, TBS Harpia = 285 spp., TBS Puma = 281 spp.). For TBS plots, Blake [74] compared the species-area accumulation curves. He found them to be virtually identical, and each approached an asymptote, indicating near-complete sampling. Of species observed at least twice, 13% were observed in one of the TBS plots but not the other. However, their absence from one plot or the other was thought to reflect real absence in most cases, not sampling error, because of the visual or auditory conspicuousness of many species in this group and inter-plot differences in microhabitat [74]. Together, these results indicate that local avian richness is fairly homogeneous throughout the park’s *terra firme* forests, and that statistical uncertainty of this richness is low. Yasuní plots [73,74] had similar sample size and effort as those in Peru [4] and French Guiana [75], making inter-site comparisons rigorous as well.

For assessments of local tree richness, 19 stand-alone 1 ha tree plots have been established in and around Yasuní [23,101], and a 50 ha CTFS plot has been established at YRS by a different research team [96]. While the stand-alone plots are distributed over a wide area, they have low standard deviation (±26 spp.) relative to their mean richness (242 spp.). In addition, the mean richness of the stand-alone plots is very similar to that documented for the CTFS plot (mean = 251 spp.) [96]. As with birds, these results indicate that tree richness is homogeneous throughout the park’s *terra firme* forests, and is likely represented accurately by existing studies. Global comparisons of local tree richness can also be made that are extremely rigorous statistically. The Yasuní CTFS plot has been censused using the same standardized methods and sampling effort that CTFS employs in other plots around the world (Table 4, references therein, and [98,229]). In addition, the stand-alone 1 ha tree plots established across the Amazon Basin are of similar enough methodology, in some cases by the same researchers, to those in Yasuní to allow for rigorous inter-Amazon comparisons [23,100,101].

However, local liana richness reports from Yasuní show that richness results may be greatly altered by sampling method, and that for some taxonomic groups there may be risks in drawing comparisons between regions, unless sample area, effort, and sampling method are all identical. Working in Yasuní and the Waorani Ethnic Reserve, Burnham established six *terra firme* liana plots of 1 ha each [95]. For each plot, five parallel, evenly spaced, but non-contiguous transects of 4 x 100 meters were used to directly sample 0.2 hectares. The plots had a low SD (mean of 98.7±9.5), suggesting that richness is fairly homogeneous throughout the park. Burnham carefully points out that the richness she documented should be taken to represent minimum richness per ha*,* not per 0.2 ha*,* because transects were non-contiguous [95]. But for rough comparisons, the Burnham plot data has been used to compare 0.1 ha liana richness globally [206]. Using a different sampling method, Nabe-Nielsen [92] established two north-south 20 x 100 m line transects in Yasuní (so each transect was 0.2 ha). He then broke down the data to correspond to four transects of 20 x 50-m, in order to compare results from 0.1 ha with others globally. These two studies of Yasuní’s liana richness had startlingly different results: 31–50 spp. documented in Burnham [95], and 12–27 species documented in Nabe-Nielsen (mean ± SD = 20.5 ± 6.2) [92]. The totals are for the same liana size classes (> 2.5 cm DBH), for samples of the same total area (0.1 ha). Yet there is no overlap between the minimum richness of the one and the maximum of the other. These and the other studies discussed above illustrate the critical need for field biologists to reach consensus on sampling protocols and statistical analyses if we are to ever fully understand global ecological and biodiversity patterns. They also illustrate the possible role that different researchers’ approaches to classifying species—such as tending to split morphologically variable collections into different species, or tending to lump them together into a single species—could play in influencing total documented richness numbers.

Our third approach to address uncertainty was to consistently note the size of sampled areas from which richness records are drawn. When studies did not give the size, we provided our own rough calculations [49]. The reader is thus alerted to any comparisons between unequal areas, providing transparency about, but not minimizing, the uncertainty of comparisons within the two spatial scales of analysis (landscape and local). In light of these three precautions and the limited state of knowledge about tropical diversity, we consider our analyses and conclusions to be as reliable and conservative as possible.

With regard to the extent and boundaries of the quadruple richness center, we acknowledge the uneven sampling across Amazonia [5,100,105,106]. In particular, areas surrounding the center’s boundaries in southern Ecuador, Colombia, and northern Peru have not been adequately studied. In addition, no data are currently available to map plant richness across South America at the local scale; the finest scale data available is 10,000 km^2^. Working within these limitations, the map of richness center overlap (Figure 3) is derived from a mixture of data, which for vertebrates is precise enough to be analyzed at a local scale (Figure 2A–C), but which for plants is at a grosser landscape scale (Figure 2D). This raises the question of whether the quadruple richness center’s location might change when global plant data become available for local-scale distributions. However, as discussed above, the most standardized and therefore definitive field inventory comparisons of Yasuní’s local richness with other sites are those for trees and shrubs (Table 4 and [23,98,100,101]), birds [72–75], and bats [85]. For all these taxa, Yasuní’s known or expected richness is among the highest in the world, substantiating the general location of the quadruple richness center. Indeed, the location of the quadruple richness center is congruent with a center of local richness for woody plants across all size classes. Thus, while the extent and boundaries of the quadruple richness center may change, we do not expect its general location to change.

**Additional Supporting Text References**

225 Bernard E, Fenton MB (2002) Species diversity of bats (Mammalia: Chiroptera) in forest fragments, primary forests, and savannas in Central Amazonia, Brazil. Canadian Journal of Zoology 80: 1124–1140.

226 Sampaio EM, Kalko EKV, Bernard E, Rodríguez HB, Handley CO Jr (2003) A biodiversity assessment of bats (Chiroptera) in a tropical lowland rainforest of Central Amazonia, including methodological and conservation considerations. Studies on Neotropical Fauna and Environment 38: 17–31.

227 Lim BK, Engstrom MD (2001) Species diversity of bats (Mammalia: Chiroptera) in Iwokrama Forest, Guyana, and the Guyanan subregion: Implications for conservation. Biodiversity Conservation 10: 613–657.

228 Simmons NB, Voss RS (1998) The mammals of Paracou, French Guyana: A Neotropical lowland rainforest fauna. Part 1. Bats. Bulletin of the American Museum of Natural History 237: 1–219.

229 Condit R, Hubbell SP, LaFrankie JV, Sukumar R, Manokaran N, *et al.* (1996) Species-area and species-individual relationships for tropical trees: A comparison of three 50 ha plots. Journal of Ecology 84: 549–562.
